# Synthesis and Evaluation of Essential Oil-Derived β-Methoxyacrylate Derivatives as High Potential Fungicides

**DOI:** 10.3390/molecules22050763

**Published:** 2017-05-08

**Authors:** Haihuan Su, Wenda Wang, Longzhu Bao, Shuangshuang Wang, Xiufang Cao

**Affiliations:** College of Science, Huazhong Agricultural University, Wuhan 430070, China; 13026346321@163.com (H.S.); hzauwangwenda@163.com (W.W.); joblongzhu@163.com (L.B.); hzaushuang@163.com (S.W.)

**Keywords:** essential oils, β-methoxyacrylate, hybrids, synthesis, fungicidal activity

## Abstract

Essential oils (EOs) are plant-derived aroma compounds with a wide range of biological activity, but their actions are slow, and they are typically unstable to light or heat, difficult to extract and so on. To find highly potential fungicides derived from natural EOs, a series of essential oil-based β-methoxyacrylate derivatives have been designed and synthesized. The target compounds have been screened for their potential fungicidal activity against eleven species of plant pathogen fungi, including *Alternaria alternata*, *Phomopsis adianticola*, *Pestalotiopsis theae*, *Sclerotinia sclerotiorum*, etc. Compared with intermediates **I**, the parent essential oils and azoxystrobin, almost all of essential oil-based β-methoxyacrylate derivatives exhibited significantly better fungicidal activity. Further investigation revealed that some compounds showed remarkable inhibitory activities against *Pestalotiopsis theae*, *Phomopsis adianticola*, *Sclerotinia sclerotiorum* and *Magnapothe grisea* at different concentrations in contrast to the commercial product azoxystrobin. Compound **II-8** exhibited particularly significant fungicidal activity.

## 1. Introduction

Essential oils (EOs) obtained by steam distillation are plant-derived aroma compounds, which are natural, lipophilic substances known for their diverse antibacterial, antifungal, anticancer, antimutagenic, antidiabetic, antiviral, antiinflammatory and antiprotozoal properties [1,2], and numerous studies during last few years have indicated that EOs and their major components exhibited non-mammalian or less mammalian toxicity according to different toxicity testing methods [1,3,4]. Besides, there are certain advantages associated with the use of EOs, including less genotoxicity, the ability to act on multiple cellular targets and low cost of production [1]. EOs possess various applications, mainly in the health, agriculture, cosmetic and food industries [5]. Many plant EOs are composed of a range of bioactive compounds that are associated with antifungal activities [2]. Muller-Riebau et al. screened nine essential oils against four species of plant pathogenic fungi including *Fusarium moniliforme*, *Rhizoctonia solani*, *Sclerotinia sclerotiorum* and *Phytophthora capsici* [6]. Wilson et al. screened 49 essential oils against the fruit pathogen *Botrytis cinerea*, and among all the 49 essential oils tested, some demonstrated the most antifungal activity against *Botrytis cinerea*, such as red thyme (*Thymus zygis*) and clove buds (*Eugenia caryophyllata*) [7]. Moreover, Mostafa and coworkers’ study confirmed that many of eleven tested plant essential oils possessed in vitro antifungal activity against *Lecanicillium fungicola* var. *fungicola* and *Agaricus bisporus* [8]. In former papers, this antifungal activity was strongly associated with the presence of monoterpenic phenols, especially thymol and carvacrol, in the essential oils [4,6,8,9,10]. Paeonol, a major phenolic component of *Paeonia suffruticosa* and *Paeonia lactiflora*, also has various biological activities [11]. However, the molecules of EOs are generally volatile, unstable to light or heat and difficult to extract, having the disadvantages of short-term fungicidal efficacy and slow action, which restrict their possible practical application.

**Figure 1:**
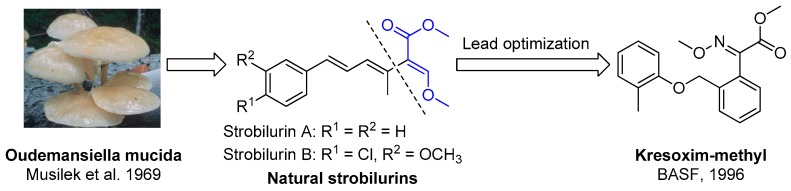
The discovery process of β-methoxyacrylates derivatives.

On the other hand, the β-methoxyacrylate unit is an important pharmacophore from natural plants, and many natural molecules with this unit have been discovered (such as the strobilurins A and B seen in Figure 1). These β-methoxyacrylate derivatives have been demonstrated to exhibit good fungicidal activities, and many such compounds have been developed as commercial fungicides (such as kresoxim-methyl, azoxystrobin, pyraclostrobin, trifloxystrobin, picoxystrobin, fluoxastrobin, orysastrobin, dimoxystrobin, coumoxystrobin, etc.). Currently, β-methoxyacrylate class is an important kind of broad-spectrum fungicides, which have a unique fungicidal mechanism, belonging to mitochondrial respiration inhibitors [12]. Kresoxim-methyl (Figure 1), which has good fungicidal activity, was the first commercial fungicide of this class [13]. However, following the commercial introduction of the strobilurin fungicides in 1996 resistant isolates were quickly detected in several plant pathogens. For example, *Erysiphe graminis* showed obvious resistance to stobilurins in Germany in 1998 [14], and, by 1999, resistant wheat isolates were found in other countries, including France, Belgium, UK and Denmark [15,16,17]. After this other resistant strains were also found successively [18,19]. Therefore, pursuing safe and eco-friendly fungicides is always a hot topic, and the research into botanical pesticides is an important aspect to prevent the emergence of resistant plant pathogenic fungi [12].

**Figure 2:**
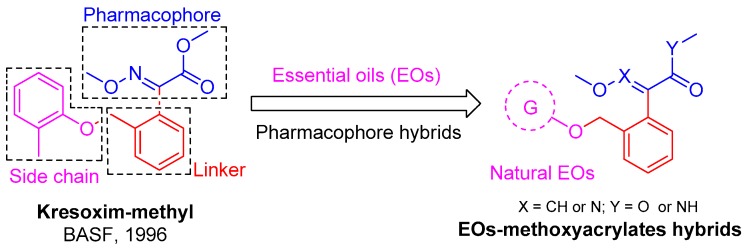
Design strategy of EOs-based β-methoxyacrylate derivatives.

During the course of our research aimed at discovering novel EO derivatives as potential agrochemicals, several series of EOs-derived chiral esters were previously investigated, which indicated their obvious insecticidal activities [20,21]. In order to improve the possibility of the application of EOs, a series of EO-methoxyacrylate derivatives have now been designed and prepared, and their potential fungicidal activities have been fully investigated. Thus, based on the aforementioned precedents, in this study natural EOs (marked in pink in Figure 2) were introduced into the strobilurin derivatives’ common skeleton. We hoped that the combination of these two moieties could result in functional synergy, and make target molecules have better biological properties, making up for the shortcomings of essential oils at the same time. Setting the kresoxim-methyl molecule as the structure model (Figure 2), we selected some phenols from essential oils to splice with the strobilurin moieties. Thereby, a series of new strobilurins derivatives have been synthesized by etherification reactions, and all newly synthesized compounds **II**, intermediates **I**, and EOs have then been screened for their potential fungicidal activity against eleven species of plant pathogen fungi.

## 2. Results and Discussion

### 2.1. Synthesis

**Scheme 1:**
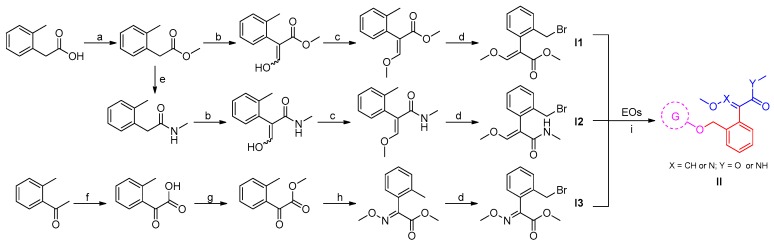
The synthetic route of EO-methoxyacrylate derivatives. *Reagents and Conditions*: (a) MeOH, H_2_SO_4_, reflux; (b) HCO_2_CH_3_, NaH, toluene, r.t.; (c) Me_2_SO_4_, K_2_CO_3_, DMF, r.t.; (d) NBS, AIBN, CCl_4_, reflux; (e) MeOH, (30–33%) methylamine water solution, reflux; (f) KMnO_4_, NaOH, H_2_O, ice water bath; (g) MeOH, H_2_SO_4_, reflux; (h) CH_3_ONH_2_-HCl, EtOH; (i) K_2_CO_3_, DMF, 80 °C, 8–12 h.

In the present study, a series of EOs-based β-methoxyacrylate derivatives were prepared via multi-step transformations. The general methods for the preparation of these EO-methoxyacrylate derivatives **II** are outlined in Scheme 1.

According to references [22,23,24], three typical β-methoxyacrylates units were selected as pharmacophores to combine with various natural EOs to investigate the effect of these substitution patterns on their fungicidal activity. First, the easily available 2-(*o*-tolyl)acetic acid was selected as starting substance, which was conveniently transferred to the corresponding intermediates **I1** and **I2** in four and five steps, respectively. For the synthesis of intermediate **I3**, the selected starting compound was 1-(*o*-tolyl)ethanone, which was first converted into the 2-oxo-2-(*o*-tolyl)acetic acid via an classic oxidation reaction, followed by a sequence including esterification, condensation and bromination reactions to afford target intermediate **I3**. Then the intermediates **I** were reacted with various EOs in the presence of a base catalyst to form the target EO-based β-methoxyacrylate derivatives **II**. All these prepared EO-methoxyacrylate derivatives **II** were obtained in medium to good yields, summarized in Table 1, and all compounds **II** and the intermediates **I** were characterized by NMR spectroscopy, MS data, and gave satisfactory chemical analyses. The chemical structures of the target compounds are also listed in Table 1.

### 2.2. Spectroscopy

**Figure 3:**
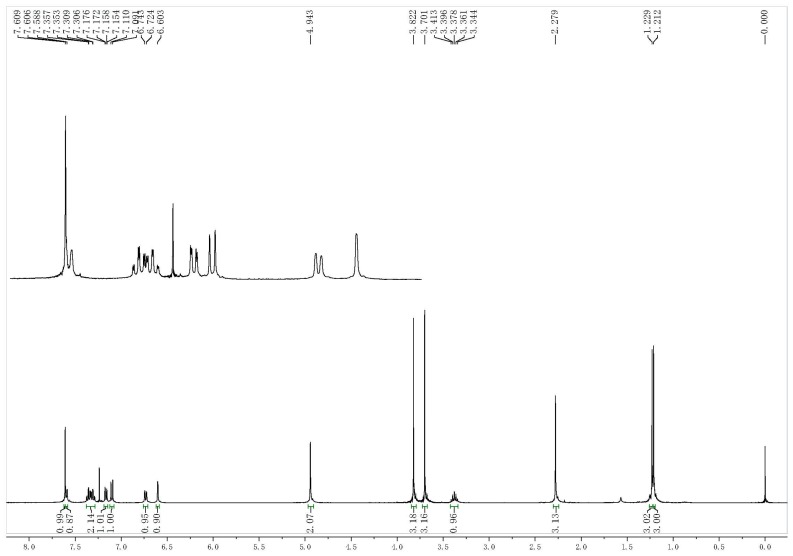
Representative ^1^H-NMR spectrum of compound **II-1**.

**Figure 4:**
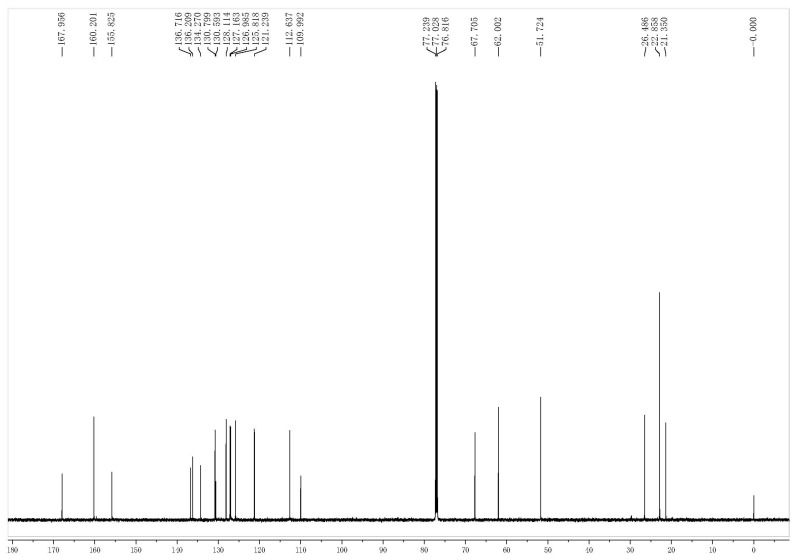
Representative ^13^C-NMR spectrum of compound **II-1**.

The analytical data for all target compounds, including ^1^H-NMR, ^13^C-NMR and ESI-MS gave satisfactory results. The ^1^H-NMR spectra of the target compounds indicated distinctive signals of aryl moiety protons, which presented several groups of peaks in the 7.88–6.39 ppm range. The general signals at higher fields in the ^1^H-NMR spectra were assigned to the alkyl protons, respectively, as shown in the representative spectrum shown in Figure 3. The ^13^C-NMR spectra presented obvious signals of different alkyl carbons (72.2–16.0), and the other signals at about 198.1–99.7 ppm in the ^13^C-NMR spectra were assigned to the C=C carbons, aromatic carbons and C=O carbons, as shown in the representative spectrum of Figure 4. The mass spectra also showed molecular ion peaks at the appropriate *m*/*z* values.

### 2.3. The In Vitro Antifungal Activity

The bioactivities of the newly prepared essential oil-based β-methoxyacrylate derivatives **II-1**~**II-17**, intermediates **I** and the corresponding essential oil molecules were all screened for their potential fungicidal activity against *Alternaria alternata, Mucor*, *Phomopsis adianticola*, *Phoma adianticola*, *Pestalotiopsis theae* and *Colletotrichum fructicola sinensis Miyake* isolated from *Camelia sinensis*; *Sclerotinia sclerotiorum* isolated from rape; *Magnapothe grisea* isolated from *Oryza sativa*; *Monilinia fructicola* isolated from peach; *Botrytis cinerea* isolated from strawberry; *Gibberella zeae* isolated from wheat at the concentration of 100 mg/L, and the bioassay primary screening results are listed in the Table 2.

Generally, as shown in Table 2, the preliminary assay illustrated that most of the synthesized EO-based β-methoxyacrylate derivatives **II** displayed good inhibitory activities against all tested fungal strains, and we also can find that most of the target compounds have better inhibitory activities than that of the corresponding intermediates **I** and the typical EO molecules (Entries 18–23) at the concentration of 100 mg/L. Notably, the inhibition rates of compounds **II-2**, **II-8**, and **II-13** on *Pestalotiopsis theae* were 92.39%, 86.45% and 93.43%, respectively, where **II-2** and **II-13** had better inhibitory activity than azoxystrobin (86.73%). Compounds **II-1**, **II-3**, and **II-13** presented 82.54%, 80.40%, and 83.77% inhibition rates against *Sclerotinia sclerotiorum*, respectively, and all exhibited obviously better inhibitory activities than the positive control azoxystrobin (31.8%). Compound **II-13** exhibited 99.72% inhibition rate against *Monilinia fructicola*, while the inhibitory activity of azoxystrobin was 96.84%*.* Compounds **II-2**, **II-3**, **II-7**, **II-8**, **II-11** and **II-13** displayed 86.55%, 82.83%, 86.12%, 82.83%, 84.19%, 95.71% fungicidal activity, respectively, against *Magnapothe grisea* and all had better inhibitory activity than azoxystrobin (83.83%), except compounds **II-3** and **II-8**. Compounds **II-13** displayed an 83.88% inhibition rate against *Gibberella zeae*, better than that of azoxystrobin (47.69%).

According to the primary screening results, compounds **II-2**, **II-8**, **II-13** had good activity against *Pestalotiopsis theae*; compounds **II-2**, **II-5**, **II-14** had good fungicidal activity against *Botrytis cinerea*; compounds **II-1**, **II-3**, **II-7**, **II-8**, **II-9**, **II-13** had good activity against *Sclerotinia sclerotiorum*; compounds **II-2**, **II-3**, **II-7**, **II-8**, **II-9**, **II-11**, **II-13** and compounds **II-2**, **II-3**, **II-5**, **II-9**, **II-10**, **II-11**, **II-12**, **II-13**, **II-14**, **II-15**, had good bioactivity against *Magnapothe grisea* and *Monilinia fructicola* respectively; and *Phomopsis adianticola* and *Gibberella zeae*, while finally compound **II-8** and compound **II-13** had better bioactivity, respectively.

Furthermore, the preliminary assay indicated many of the target compounds exhibited good fungicidal activities compared to the commercial fungicide azoxystrobin (Table 2), so in order to further investigate the potential fungicidal activities, the efficient concentration values (EC_50_) for typical compounds were used to further evaluate the fungicidal activity at different concentrations, including 6.25, 12.5, 25, 50 and 100 mg/L. The fungicidal activities expressed as EC_50_ values for highly potential compounds are listed in Table 3, and further prove that some of these EO-based β-methoxyacrylate derivatives **II** exhibited higher fungicidal activity than the commercial compound azoxystrobin under the same conditions.

As shown in Table 3, the EC_50_ values indicated that many target compounds had better fungicidal activity compared with the positive control azoxystrobin. For *Pestalotiopsis theae*, *Phomopsis adianticola*, *Sclerotinia sclerotiorum* and *Magnapothe grisea* the EC_50_ values of compound **II-8**, which were 0.04, 3.06, 0.003 nd 0.95 mg/L, respectively, were the smallest; for *Gibberella zeae*, compound **II-13** with an EC_50_ value of 15.48 mg/L had better fungicidal activity than azoxystrobin. On the whole, the compound **II-8** exhibited obviously better activity against *Pestalotiopsis theae*, *Phomopsis adianticola*, *Sclerotinia sclerotiorum* and *Magnapothe grisea* than azoxystrobin.

**Figure 5:**
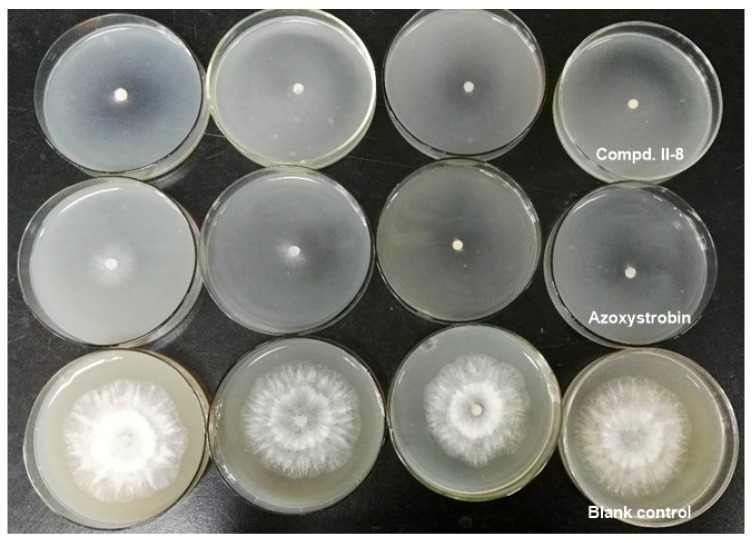
Mycelial growth of *Pestalotiopsis theae* using compound **II-8** and azoxystrobin at the different concentration of 6.25, 12.5, 25 and 50 mg/L.

In addition, the effects of the compound **II-8**, azoxystrobin and blank control on mycelial growth of *Pestalotiopsis theae* were investigated. As shown in Figure 5, under the desired concentration of 6.25 mg/L, 12.5 mg/L, 25 mg/L and 50 mg/L, the diameter ± SD of inhibition zone reached 18.50 ± 0.71, 12.23 ± 0.99, 10.50 ± 0.71 and 9.75 ± 2.12 mm; 25.68 ± 0.60, 17.08 ± 5.06, 11.25 ± 1.06 and 10.15 ± 1.56 mm; 61.90 ± 0.14, 60.55 ± 0.78, 57.00 ± 2.47 and 61.98 ± 0.11 mm, respectively. Compound **II-8** presented significant inhibition compared to the positive control azoxystrobin and blank control.

According to the above results, considering the EO portion, when natural essential oil molecules such as thymol and carvacrol were introduced, many compounds indicated wonderful activity, which maybe because thymol and carvacrol themselves display fungicidal activity. In addition, considering the strobilurin derivatives’ common skeleton, compounds indicated better fungicidal activity when an (*E*)-carboxamide methoxypropenoate acrylate unit was introduced into the molecules instead of an (*E*)-methoxyacrylate, so maybe the presence of N atoms allows the N-H bond to form hydrogen bonds with other electron-rich atoms or groups (such as O, F, N, or benzene rings), which could improve the bioactivity.

## 3. Experimental

### 3.1. General Information

Melting points (m.p.) were determined on an X-4 digital display microscopic melting point apparatus (Shanghai Instrument Physical Optics Instrument Co., Ltd., Shanghai, China). ^1^H-NMR spectra were recorded on a Bruker spectrometer at 600 or 400 MHz (Bruker, Bremen, Germany) with CDCl_3_ as the solvent and TMS as the internal standard; ^13^C-NMR spectra were recorded on a Bruker spectrometer at 150 MHz with CDCl_3_ as the solvent. Chemical shifts are reported in *δ* (parts per million) values. Coupling constants ^n^*J* are reported in Hz. Mass spectra were performed on a Waters ACQUITY UPLC^®^ H-CLASS PDA (Waters^®^, Milford, MA, USA) instrument. Analytical thin-layer chromatography (TLC) was carried out on pre-coated plates, and spots were visualized with ultraviolet light. Column chromatography was carried out on silica gel (200–300 mesh, Qingdao Haiyang Chemical, Qingdao, China). Agar (Shanghai Regal Chemical, Shanghai, China), d-(+)-glucose (Shanghai Hushi Chemical, Shanghai, China). All other solvents and reagents were analytical reagent and used directly without purification.

### 3.2. General Synthetic Procedure for Intermediates ***I***

2-(*o*-Tolyl)acetic acid or 1-(*o*-tolyl)ethanone as starting materials were prepared according to the patent literature [22,23,24]. Intermediates **I** were synthesized via esterification, ester condensation, methylation, and bromination reactions. All intermediates **I** (Scheme 1) have satisfactory chemical analytical data.

### 3.3. General Synthetic Procedure for Target Compounds ***II***

A mixture of essential oil phenol (2.11 mmol) and anhydrous potassium carbonate (2.64 mmol) in DMF (8 mL) was stirred at room temperature for 0.5 h. Then intermediates **I** (1.76 mmol) were added and the reaction mixtures were stirred for 8–12 h at 80 °C. The resulting mixtures were cooled to room temperature and filtered, and then the reaction mixtures were extracted with ethyl acetate (3 × 10 mL). The combined organic layers were washed with brine (3 × 20 mL), dried over anhydrous sodium sulfate and concentrated with a rotary evaporator. The crude products were purified by chromatography on silica using a mixture of petroleum ether and ethyl acetate as an eluent to give the target essential oils-oriented β-methoxyacrylate derivatives **II**. All the characterization data are as follows and the corresponding spectra of the target molecules are presented in the Supplementary Materials.

*(E)-2-(2-((2-Isopropyl-5-methyl)phenoxymethyl)phenyl)-3-methoxyacrylate* (**II-1**). This compound was obtained as a yellowish solid following the abovementioned method, m.p. 76–77 °C. ^1^H-NMR (400 MHz, CDCl_3_): δ = 7.61 (s, 1H), 7.59 (d, *J* = 4 Hz, 1H), 7.37–7.29 (m, 2H), 7.16 (dd, 1H), 7.10 (d, *J* = 4 Hz, 1H), 6.73 (d, *J* = 8 Hz, 1H), 6.60 (s, 1H), 4.94 (s, 2H), 3.82 (s, 3H), 3.70 (s, 3H), 3.41–3.34 (m, 1H), 2.28 (s, 3H), 1.23 (s, 3H), 1.21 (s, 3H); ^13^C-NMR (150 MHz, CDCl_3_): δ = 168.0, 160.2, 155.8, 136.7, 136.2, 134.3, 130.8, 130.6, 128.1, 127.2, 127.0, 125.8, 121.2, 112.6, 110.0, 67.7, 62.0, 51.7, 26.5, 22.8, 21.4; MS (ESI) *m*/*z* 377.4 (M + Na)^+^, calcd. for C_22_H_26_O_4_ *m*/*z* = 354.2.

*(E)-2-(2-((5-Isopropyl-2-methyl)phenoxymethyl)phenyl)-3-methoxyacrylate* (**II-2**). This compound was obtained as a yellowish solid following the abovementioned method, m.p. 66–67 °C. ^1^H-NMR (400 MHz, CDCl_3_): δ = 7.60 (s, 1H), 7.58 (d, *J* = 8 Hz, 1H), 7.36–7.29 (m, 2H), 7.17 (dd, 1H), 7.06 (d, *J* = 8Hz, 1H), 6.75–6.71 (m, 1H), 6.62 (s, 1H), 4.96 (s, 2H), 3.82 (s, 3H), 3.70 (s, 3H), 2.96–2.77 (m, 1H), 2.23 (s, 3H), 1.20 (s, 3H), 1.18 (s, 3H); ^13^C-NMR (150 MHz, CDCl_3_): δ = 168.0, 160.1, 156.8, 147.8, 136.6, 130.8, 130.7, 130.4, 128.0, 127.2, 124.3, 117.9, 110.0, 67.7, 61.9, 51.7, 34.1, 24.1, 16.0; MS (ESI) *m*/*z* 377.3 (M + Na)^+^, calcd. for C_22_H_26_O_4_ *m*/*z* = 354.2.

*(E)-2-(2-((4-Allyl-2-methoxy)phenoxymethyl)phenyl)-3-methoxyacrylate* (**II-3**). This compound was obtained as a yellowish liquid following the abovementioned method, ^1^H-NMR (400 MHz, CDCl_3_): δ = 7.59 (s, 1H), 7.56 (d, *J* = 8 Hz, 1H), 7.32–7.26 (m, 2H), 7.14 (dd, 1H), 6.71 (s, 1H), 6.66 (d, *J* = 8 Hz, 1H), 6.58 (dd, 1H), 5.98–5.88 (m, 1H), 5.09–5.04 (m, 4H), 3.88 (s, 3H), 3.82 (s, 3H), 3.70 (s, 3H), 3.30 (d, *J* = 8 Hz, 2H); ^13^C-NMR (150 MHz, CDCl_3_): δ = 168.0, 160.1, 149.4, 146.5, 137.7, 136.4, 132.9, 130.8, 130.5, 128.1, 127.1, 126.8, 120.4, 115.6, 113.9, 112.3, 109.9, 68.8, 62.0, 55.9, 51.7, 39.8; MS (ESI) *m*/*z* 391.4 (M + Na)^+^, calcd. for C_22_H_24_O_5_ *m*/*z* = 368.2.

*(E)-2-((2-Carboxylate-phenoxymethyl)phenyl)-3-methoxyacrylate* (**II-4**). This compound was obtained as a white solid following the abovementioned method, m.p. 112–113 °C. ^1^H-NMR (400 MHz, CDCl_3_): δ = 7.79 (dd, 1H), 7.70 (d, *J* = 8 Hz, 1H), 7.61 (s, 1H), 7.36–7.27 (m, 3H), 7.15 (dd, 1H), 6.96–6.93 (m, 1H), 6.86 (d, *J* = 8 Hz, 1H), 5.09 (s, 2H), 3.92 (s, 3H), 3.82 (s, 3H), 3.70 (s, 3H); ^13^C-NMR (150 MHz, CDCl_3_): δ = 167.8, 166.9, 160.2, 158.0, 135.9, 133.3, 131.6, 130.8, 130.2, 128.2, 127.2, 126.6, 120.5, 120.2, 113.7, 109.8, 68.3, 62.0, 52.0, 51.7; MS (ESI) *m*/*z* 379.3 (M + Na)^+^, calcd. for C_20_H_20_O_6_ *m*/*z* = 356.1.

*(E)-2-(2-((2-Acetyl-4-methoxy)phenoxymethyl)phenyl)-3-methoxyacrylate* (**II-5**). This compound was obtained as a yellowish solid following the abovementioned method, m.p. 126–127 °C. ^1^H-NMR (600 MHz, CDCl_3_): δ = 7.82 (d, *J* = 6 Hz, 1H), 7.61 (s, 1H), 7.52 (t, *J*_1_ = 3.6 Hz, *J*_2_ = 6 Hz, 1H), 7.36–7.32 (m, 2H), 7.20–7.18 (m, 1H), 6.50 (dd, 1H), 6.40 (d, *J* = 2.4 Hz, 1H), 5.06 (s, 2H), 3.81 (s, 3H), 3.78 (s, 3H), 3.70 (s, 3H), 2.57 (s, 3H); ^13^C-NMR (150 MHz, CDCl_3_): δ = 198.1, 167.7, 164.4, 160.4, 160.2, 135.2, 132.6, 131.2, 128.2, 127.8, 127.6, 121.4, 109.8, 105.1, 99.8, 68.6, 62.0, 55.5, 51.7, 32.1; MS (ESI) *m*/*z* 393.4 (M + Na)^+^, calcd. for C_21_H_22_O_6_ *m*/*z* = 370.1.

*(E)-2-(2-(2,6-Dimethoxyphenoxymethyl)phenyl)-3-methoxyacrylate* (**II-6**). This compound was obtained as a yellow solid following the abovementioned method, m.p. 87–88 °C. ^1^H-NMR (600 MHz, CDCl_3_): δ = 7.88 (d, *J* = 12 Hz, 1H), 7.56 (s, 1H), 7.36 (t, *J*_1_ = 6 Hz, *J*_2_ = 12 Hz, 1H), 7. 26 (t, *J*_1_ = *J*_2_ = 6 Hz, 1H), 7.12 (d, *J* = 12 Hz, 1H), 6.96 (t, *J*_1_ = 6 Hz, *J*_2_ = 12 Hz, 1H), 6.56 (d, *J* = 6 Hz, 2H), 4.88 (s, 2H), 3.79 (s, 6H), 3.74 (s, 3H), 3.64 (s, 3H); ^13^C-NMR (150 MHz, CDCl_3_): δ = 168.1, 160.1, 153.9, 137.5, 137.3, 131.0, 130.4, 128.4, 127.8, 127.1, 123.6, 110.1, 105.5, 72.2, 61.8, 56.1, 51.5; MS (ESI) *m*/*z* 381.3 (M + Na)^+^, calcd. for C_20_H_22_O_6_ *m*/*z* = 358.1.

*(E)-2-(2-((2-Isopropyl-5-methyl)phenoxymethyl)phenyl)-3-methoxy-N-methylacrylamide* (**II-7**). This compound was obtained as yellowish solid following the abovementioned method, m.p. 73–74 °C. ^1^H-NMR (400 MHz, CDCl_3_): δ = 7.62 (s, 1H), 7.60 (s, 1H), 7.39–7.30 (m, 2H), 7.18 (dd, 1H), 7.12 (d, *J* = 8 Hz, 1H), 6.75 (d, *J* = 8 Hz, 1H), 6.62 (s, 1H), 4.96 (s, 2H), 3.84 (s, 3H), 3.72 (s, 3H), 3.43–3.36 (m, 1H), 2.29 (s, 3H), 1.25 (s, 3H), 1.23 (s, 3H); ^13^C-NMR (150 MHz, CDCl_3_): δ = 168.0, 160.2, 155.8, 136.7, 136.2, 134.3, 130.8, 130.6, 128.1, 127.2, 127.0, 125.8, 121.2, 112.6, 110.0, 67.7, 62.0, 51.7, 26.5, 22.9, 21.4; MS (ESI) *m*/*z* 377.4 (M + Na + H)^+^, calcd. for C_22_H_27_ NO_3_ *m*/*z* = 353.2.

*(E)-2-(2-((5-Isopropyl-2-methyl)phenoxymethyl)phenyl)-3-methoxy-N-methylacrylamide* (**II-8**). This compound was obtained as a yellowish solid following the abovementioned method, m.p. 76–77 °C. ^1^H-NMR (400 MHz, CDCl_3_): δ = 7.60 (s, 1H), 7.58 (d, *J* = 4 Hz, 1H), 7.37-7.29 (m, 2H), 7.18 (dd, 1H), 7.06 (d, *J* = 8 Hz, 1H), 6.72 (dd, 1H), 6.62 (s, 1H), 4.97 (s, 2H), 3.82 (s, 3H), 3.70 (s, 3H), 2.85–2.78 (m, 1H), 2.24 (s, 3H), 1.21 (s, 3H), 1.19 (s, 3H); ^13^C-NMR (150 MHz, CDCl_3_): δ = 168.0, 160.1, 156.8, 147.8, 136.6, 130.9, 130.7, 130.4, 128.0, 127.2, 124.3, 117.9, 110.0, 67.7, 62.0, 51.7, 34.1, 24.1, 16.0; MS (ESI) *m*/*z* 377.4 (M + Na + H)^+^, calcd. for C_22_H_27_NO_3_ *m*/*z* = 353.2.

*(E)-2-(2-((4-Allyl-2-methoxy)phenoxymethyl)phenyl)-3-methoxy-N-methylacrylamide* (**II-9**). This compound was obtained as a yellow liquid following the abovementioned method, ^1^H- NMR (400 MHz, CDCl_3_): δ = 7.59 (s, 1H), 7.55 (d, *J* = 4 Hz, 1H), 7.32–7.26 (m, 2H), 7.14 (dd, 1H), 6.71 (s, 1H), 6.66 (d, *J* = 4 Hz, 1H), 6.58 (dd, 1H), 5.98–5.88 (m, 1H), 5.09–5.04 (m, 4H), 3.88 (s, 3H), 3.82 (s, 3H), 3.70 (s, 3H), 3.30 (d, *J* = 8 Hz, 2H); ^13^C-NMR (150 MHz, CDCl_3_): δ = 167.9, 160.1, 149.4, 146.5, 137.7, 136.4, 132.9, 130.8, 130.5, 128.1, 127.1, 126.8, 120.4, 115.6, 113.9, 112.3, 109.9, 68.8, 62.0, 55.9, 51.7, 39.8; MS (ESI) *m*/*z* 391.4 (M + Na + H)^+^, calcd. for C_22_H_25_ NO_4_ *m*/*z* = 367.2.

*(E)-2-((2-Carboxylatephenoxymethyl)phenyl)-3-methoxy-N-methylacrylamide* (**II-10**). This compound was obtained as a white solid following the abovementioned method, m.p. 105–106 °C. ^1^H-NMR (400 MHz, CDCl_3_): δ = 7.79 (dd, 1H), 7.70 (d, *J* = 8 Hz, 1H), 7.61 (s, 1H), 7.37–7.27 (m, 3H), 7.15 (d, *J* = 8 Hz, 1H), 6.94 (t, *J*_1_ *= J*_2_ = 8 Hz, 1H), 6.86 (d, *J* = 8 Hz, 1H), 5.09 (s, 2H), 3.91 (s, 3H), 3.82 (s, 3H), 3.70 (s, 3H); ^13^C-NMR (150 MHz, CDCl_3_): *δ* = 167.8, 166.9, 160.2, 158.1, 135.9, 133.3, 131.7, 130.8, 130.2, 128.2, 127.2, 126.6, 120.6, 120.3, 113.7, 109.8, 68.3, 62.0, 52.0, 51.7; MS (ESI) *m*/*z* 379.4 (M + Na + H)^+^, calcd. for C_20_H_21_NO_5_ *m*/*z* = 355.1.

*(E)-2-(2-((2-Acetyl-4-methoxy)phenoxymethyl)phenyl)-3-methoxy-N-methylacrylamide* (**II-11**). This compound was obtained as a yellowish solid following the abovementioned method, m.p. 122–123 °C. ^1^H-NMR (600 MHz, CDCl_3_): δ = 7.82 ( d, *J* = 6 Hz, 1H), 7.60 (s, 1H), 7.51 (t, *J*_1_ = 3 Hz, *J*_2_ = 6 Hz, 1H), 7.35–7.32 ( m, 2H), 7.20–7.18 (m, 1H), 6.49 (d, *J* = 6 Hz, 1H), 6.39 (d, *J* = 6 Hz, 1H), 5.06 (s, 2H), 3.81 (s, 3H), 3.77 (s, 3H), 3.69 (s, 3H), 2.57 (s, 3H); ^13^C-NMR (150 MHz, CDCl_3_): δ = 198.0, 167.7, 164.4, 160.2, 135.2, 132.6, 131.2, 128.2, 127.8, 127.5, 121.4, 109.8, 105.1, 99.8, 68.6, 62.0, 55.4, 51.7, 32.1; MS (ESI) *m*/*z* 393.4 (M + Na + H)^+^, calcd. for C_21_H_23_NO_5_ *m*/*z* = 369.2.

*(E)-2-(2-(2,6-Dimethoxyphenoxymethyl)phenyl)-3-methoxy-N-methylacrylamide* (**II-12**). This compound was obtained as a yellow solid following the abovementioned method, m.p. 83–84 °C. ^1^H-NMR (600 MHz, CDCl_3_): δ = 7.88 (d, *J* = 6 Hz, 1H), 7.55 (s, 1H), 7.37 (t, *J*_1_ = *J*_2_ = 6 Hz, 1H), 7. 28 (t, *J*_1_ = 6 Hz, *J*_2_ = 12 Hz, 1H), 7.12 (d, *J* = 6 Hz, 1H), 6.98 (t, *J*_1_ = 12 Hz, *J*_2_ = 6 Hz, 1H), 6.56 (d, *J* = 6 Hz, 2H), 4.88 (s, 2H), 3.80 (s, 6H), 3.77 (s, 3H), 3.65 (s, 3H). ^13^C-NMR (150 MHz, CDCl_3_): δ = 168.2, 160.1, 153.9, 137.5, 137.3, 131.0, 130.4, 128.5, 127.8, 127.1, 123.6, 110.1, 105.5, 72.2, 61.8, 56.1, 51.5; MS (ESI) *m*/*z* 381.4 (M + Na + H)^+^, calcd. for C_20_H_23_NO_5_ *m*/*z* = 357.2.

*(E)-2-(2-((2-Isopropyl-5-methyl)phenoxymethyl)phenyl)-2-(methoxyimino)acetate* (**II-13**). This compound was obtained as a yellowish solid following the above mentioned method, m.p. 80–81 °C. ^1^H-NMR (600 MHz, CDCl_3_): δ = 7.62 (d, *J* = 6 Hz, 1H), 7.46 (t, *J*_1_ = 12 Hz, *J*_2_ = 6 Hz, 1H), 7.38 (t, *J*_1_ = *J*_2_ = 6 Hz, 1H), 7.20 (d, *J* = 12 Hz, 1H), 7.10 (d, *J* = 6 Hz, 1H), 6.76 (d, *J* = 6 Hz, 1H), 6.62 (s, 1H), 4.92 (s, 2H), 4.05 (s, 3H), 3.84 (s, 3H), 3.36–3.31 (m, 1H), 2.29 (s, 3H), 1.20 (d, *J* = 6 Hz, 6H); ^13^C-NMR (150 MHz, CDCl_3_): δ = 163.3, 155.5, 149.3, 136.3, 135.9, 134.3, 129.7, 128.7, 128.3, 127.4, 125.9, 121.6, 112.7, 68.0, 63.9, 53.0, 26.4, 22.9, 21.3; MS (ESI) *m*/*z* 378.4 (M + Na)^+^, calcd. for C_21_H_25_NO_4_ *m*/*z* = 355.2.

*(E)-2-(2-((4-Allyl-2-methoxy)phenoxymethyl)phenyl)-2-(methoxyimino)acetate* (**II-14**). This compound was obtained as a yellowish solid following the abovementioned method, m.p. 48–49 °C. ^1^H-NMR (600 MHz, CDCl_3_): δ = 7.58 (d, *J* = 6 Hz, 1H), 7.40 (t, *J*_1_ = *J*_2_ = 6 Hz, 1H), 7.34 (t, *J*_1_ = 12 Hz, *J*_2_ = 6 Hz, 1H), 7.16 (d, *J* = 6 Hz, 1H), 6.71 (d, *J* = 12 Hz, 2H), 6.62 (d, *J* = 12 Hz, 1H), 5.97-5.90 (m, 1H), 5.06 (t, *J*_1_ = 18 Hz, *J*_2_ = 6 Hz, 2H), 4.98 (s, 2H), 4.02 (s, 3H), 3.85 (s, 3H), 3.84 (s, 3H), 3.30 (d, *J* = 6 Hz, 2H); ^13^C-NMR (150 MHz, CDCl_3_): δ = 163.3, 149.6, 149.4, 146.3, 137.6, 135.7, 133.5, 129.6, 129.0, 128.2, 127.5, 120.4, 115.6, 114.3, 112.5, 69.2, 63.8, 55.9, 52.9, 39.8; MS (ESI) *m*/*z* 392.4 (M + Na)^+^, calcd. for C_21_H_23_NO_5_ *m*/*z* = 369.2.

*(E)-2-((2-Carboxylate phenoxymethyl)phenyl)-2-(methoxyimino)acetate* (**II-15**). This compound was obtained as yellowish solid following the abovementioned method, m.p. 100–101 °C. ^1^H-NMR (600 MHz, CDCl_3_): δ = 7.81 (dd, 1H), 7.74 (d, *J* = 6 Hz, 1H), 7.46 (t, *J*_1_ = 6 Hz, *J*_2_ = 12 Hz, 1H), 7.40–7.36 (m, 2H), 7.18 (d, *J* = 6 Hz, 1H), 6.98 (t, *J*_1_ = 6 Hz, *J*_2_ = 12 Hz, 1H), 6.90 (d, *J* = 6 Hz, 1H), 5.05 (s, 2H), 4.03 (s, 3H), 3.90 (s, 3H), 3.85 (s, 3H); ^13^C-NMR (150 MHz, CDCl_3_): δ = 166.7, 163.2, 157.8, 149.2, 135.1, 133.4, 131.8, 129.7, 128.4, 128.2, 127.5, 127.1, 120.6, 113.7, 68.5, 63.8, 53.0, 52.0; MS (ESI) *m*/*z* 380.4 (M + Na)^+^, calcd. for C_19_H_19_NO_6_ *m*/*z* = 357.1.

*(E)-2-(2-((2-Acetyl-4-methoxy)phenoxymethyl)phenyl)-2-(methoxyimino)acetate* (**II-16**). This compound was obtained as a light pink solid following the abovementioned method, m.p. 97–98 °C. ^1^H-NMR (600 MHz, CDCl_3_): δ = 7.83 (d, *J* = 12 Hz, 1H), 7.56 (d, *J* = 6 Hz, 1H), 7.46 (t, *J*_1_ = 12 Hz, *J*_2_ = 6 Hz,1H), 7.42 (t, *J*_1_ = *J*_2_ = 6 Hz, 1H), 7.22 (d, *J* = 6 Hz, 1H), 6.52 (dd, 1H), 6.40 (d, *J* = 1.8 Hz, 1H), 5.02 (s, 2H), 4.04 (s, 3H), 3.83 (s, 3H), 3.79 (s, 3H), 2.54 (s, 3H); ^13^C-NMR (150 MHz, CDCl_3_): δ = 197.8, 164.3, 163.2, 159.8, 149.0, 134.4, 132.7, 129.8, 129.2, 128.6, 128.1, 127.8, 121.4, 105.3, 99.7, 68.6, 63.9, 55.5, 53.1, 32.0; MS (ESI) *m*/*z* 394.4 (M + Na)^+^, calcd. for C_20_H_21_NO_6_ *m*/*z* = 371.1.

*(E)-2-(2-(2,6-Dimethoxyphenoxymethyl)phenyl)-2-(methoxyimino)acetate* (**II-17**). This compound was obtained as a yellowish solid following the abovementioned method, m.p. 103–104 °C. ^1^H-NMR (600 MHz, CDCl_3_): δ = 7.88 (d, *J* = 12 Hz, 1H), 7.46 (t, *J*_1_ = *J*_2_ = 6 Hz, 1H), 7.36 (t, *J*_1_ = *J*_2_ = 6 Hz, 1H), 7.14 (d, *J* = 6 Hz, 1H), 6.98 (t, *J*_1_ = 6 Hz, *J*_2_ = 12 Hz, 1H), 6.56 (d, *J* = 6 Hz, 2H), 4.83 (s, 2H), 4.00 (s, 3H), 3.82 (s, 3H), 3.80 (s, 6H); ^13^C-NMR (150 MHz, CDCl_3_): δ = 163.3, 153.8, 149.3, 137.0, 136.4, 129.4, 129.2, 129.0, 127.8, 127.4, 123.8, 105.3, 72.1, 63.7, 56.0, 52.8; MS (ESI) *m*/*z* 382.5 (M + Na)^+^, calcd. for C_19_H_21_NO_6_ *m*/*z* = 359.1.

### 3.4. Biological Assays

The fungi used in this study include *Alternaria alternata*, *Mucor*, *Phomopsis adianticola*, *Phoma adianticola*, *Pestalotiopsis theae* and *Colletotrichum fructicola sinensis* Miyake isolated from *Camellia sinensis*; *Sclerotinia sclerotiorum* isolated from rape; *Magnapothe grisea* isolated from *Oryza sativa*; *Monilinia fructicola* isolated from peach; *Botrytis cinerea* isolated from strawberry; *Gibberella zeae* isolated from wheat as well as. Target compounds **II**, intermediates **I** and essential oil molecules were diluted in acetone and added to Potato Dextrose Agar (PDA) culture medium according to desired final concentrations [2,8,9], pouring into Petri plates (9-mm diameter). Each sample was screened at a concentration of 100 mg/L, after that further evaluation was performed based on the a preliminary results, involving 6.25, 12.5, 25, 50 and 100 mg/L, acetone solutions (less than 1% *v*/*v* [9]). Then, each dish was inoculated in the center with a 5 mm diameter stipe, containing mycelia of the fungus culture. Positive control sets were run simultaneously, using the medium containing azoxystrobin, and which is a representative strobilurin fungicide [12,17], and blank control consisted of unamended PDA medium supplemented with the corresponding concentration of acetone. Dishes were sealed and incubated at a temperature of 27 ± 1 °C for four to six days in a culture chamber, and then diameter mycelium was measured. The percentage of relative inhibition was computed after comparison with the blank control using the following formula [25]:

(1) I=[(C−T)/C]×100

where *I* is growth inhibition (%), C and T represent mycelial growth diameter in control and target compounds **II**, intermediates **I** and essential oil molecules in the treated Petri plates, respectively.

EC_50_ values (mg/L concentrations inhibiting radial mycelial growth by 50%) for the studied compounds were determined by interpolation from computer-generated log-probit plots of compounds concentrations and relative inhibition (Probit SPSS version 22).

## 4. Conclusions

In summary, seventeen new essential oil-based β-methoxyacrylate derivatives have been designed, synthesized and evaluated as potential fungicides. The structures of all obtained molecules were characterized by ^1^H-NMR, ^13^C-NMR and ESI-MS spectra analyses. The preliminary bioassays indicated that almost of target compounds exhibited significant fungicidal activity compared with the commercial compound azoxystrobin, and some compounds presented good fungicidal activity at lower concentrations. These results also demonstrated that the combination of β-methoxyacrylate units with essential oils could lead to new active molecules, which may have potential application for plant fungi treatment. Further structural optimization and specific structure-activity relationship studies are still under investigation in our laboratory.

## Figures and Tables

**Table 1:** The chemical structure of target compounds **II-1**~**II-17**.

Compd. No.	Substituents			Appearance	m.p. (°C)	Yield (%) ^a^
	G	X	Y			
**II-1**	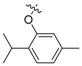	CH	O	Yellowish solid	76–77	42.98
**II-2**	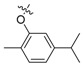	CH	O	Yellowish solid	66–67	78.50
**II-3**	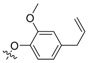	CH	O	Yellowish liquid	-	37.72
**II-4**	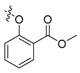	CH	O	White solid	112–113	82.83
**II-5**	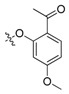	CH	O	Yellowish solid	126–127	38.40
**II-6**	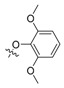	CH	O	Yellow solid	87–88	56.56
**II-7**	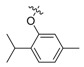	CH	NH	Yellowish solid	73–74	37.60
**II-8**	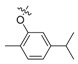	CH	NH	Yellowish solid	76–77	47.28
**II-9**	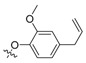	CH	NH	Yellow liquid	-	31.68
**II-10**	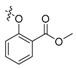	CH	NH	White solid	105–106	90.72
**II-11**	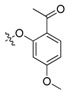	CH	NH	Yellowish solid	122–123	37.60
**II-12**	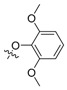	CH	NH	Yellow solid	83–84	62.00
**II-13**	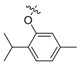	N	O	Yellowish solid	80–81	70.23
**II-14**	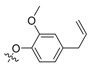	N	O	Yellowish solid	48–49	74.54
**II-15**	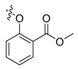	N	O	Yellowish solid	100–101	83.12
**II-16**	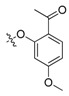	N	O	Light pink solid	97–98	81.83
**II-17**	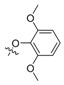	N	O	Yellowish solid	103–104	68.02

^a^ Isolated yield.

**Table 2:** The fungicidal activity of EOs-oriented β-methoxyacrylates derivatives **II-1**~**II-17** at a concentration of 100 mg/L.

Entry	Compd. No.	Inhibition Activity (%)										
		*Pestalotiopsis theae*	*Botrytis cinerea*	*Colletotrichum fructicola sinensis Miyake*	*Sclerotinia sclerotiorum*	*Mucor*	*Phoma adianticola*	*Monilinia fructicola*	*Alternaria alternata*	*Phomopsis adianticola*	*Magnapothe grisea*	*Gibberella zeae*
1	**II-1**	75.50 ± 4.45	57.50 ± 4.82	54.23 ± 3.52	82.54 ± 4.66	44.02 ± 1.98	60.23 ± 1.38	70.35 ± 8.02	38.98 ± 1.03	64.22 ± 1.23	74.39 ± 4.86	15.48 ± 0.00
2	**II-2**	92.39 ± 1.30	66.73 ± 13.56	58.94 ± 2.24	70.43 ± 10.60	48.99 ± 3.35	70.99 ± 0.23	92.29 ± 7.23	47.33 ± 1.83	64.14 ± 7.15	86.55 ± 0.61	44.13 ± 0.34
3	**II-3**	71.39 ± 2.03	45.59 ± 2.76	53.92 ± 0.67	80.40 ± 1.40	46.86 ± 1.37	68.14 ± 0.81	90.89 ± 1.05	38.98 ± 0.57	52.37 ± 2.57	82.83 ± 0.00	34.72 ± 3.50
4	**II-4**	51.71 ± 5.18	42.77 ± 3.15	35.66 ± 2.24	65.82 ± 19.22	31.99 ± 0.11	19.65 ± 3.92	64.41 ± 2.23	17.10 ± 3.09	55.61 ± 3.13	46.92 ± 2.43	14.13 ± 5.87
5	**II-5**	60.16 ± 3.61	76.14 ± 2.12	38.62 ± 7.48	72.98 ± 2.10	51.24 ± 6.15	31.96 ± 5.88	85.51 ± 3.55	44.98 ± 4.01	53.08 ± 0.22	47.28 ± 6.78	22.35 ± 3.50
6	**II-6**	45.42 ± 2.48	17.73 ± 0.00	33.49 ± 0.52	54.37 ± 9.79	28.53 ± 1.81	28.94 ± 1.84	80.39 ± 4.34	38.49 ± 3.09	41.07 ± 10.50	65.45 ± 2.33	35.67 ± 8.92
7	**II-7**	76.93 ± 6.70	48.27 ± 2.31	41.27 ± 7.33	79.57 ± 3.73	41.03 ± 2.25	51.11 ± 0.23	78.16 ± 9.59	33.95 ± 0.11	42.26 ± 5.77	86.12 ± 0.81	36.27 ± 1.19
8	**II-8**	86.45 ± 1.69	54.91 ± 3.86	50.58 ± 1.95	75.04 ± 4.31	47.71 ± 1.70	65.94 ± 1.84	81.97 ± 19.45	49.11 ± 0.23	68.72 ± 2.23	82.83 ± 1.01	38.51 ± 3.56
9	**II-9**	73.43 ± 0.17	41.18 ± 10.03	53.92 ± 0.67	76.85 ± 1.05	57.96 ± 7.52	69.77 ± 1.27	91.08 ± 1.05	52.19 ± 0.00	52.76 ± 2.01	77.90 ± 4.96	31.36 ± 3.84
10	**II-10**	45.10 ± 5.40	48.91 ± 0.00	31.85 ± 3.44	63.34 ± 14.56	44.29 ± 4.67	16.88 ± 2.07	87.55 ± 1.84	39.06 ± 2.52	45.34 ± 14.30	60.80 ± 15.58	6.86 ± 8.24
11	**II-11**	55.26 ± 4.90	64.41 ± 0.19	41.85 ± 5.91	72.16 ± 4.66	48.06 ± 0.33	38.80 ± 0.81	87.73 ± 3.15	50.24 ± 1.38	49.37 ± 2.79	84.19 ± 0.10	19.63 ± 7.22
12	**II-12**	49.44 ± 0.06	24.27 ± 0.00	36.19 ± 4.49	67.13 ± 0.12	32.88 ± 2.91	31.06 ± 3.23	95.54 ± 3.42	41.25 ± 1.03	43.05 ± 5.25	70.17 ± 0.91	48.40 ± 0.06
13	**II-13**	93.43 ± 1.41	82.00 ± 5.40	55.77 ± 8.83	83.77 ± 0.35	67.24 ± 8.45	58.77 ± 0.46	99.72 ± 0.39	62.56 ± 1.38	42.02 ± 6.70	95.71 ± 1.01	83.88 ± 8.80
14	**II-14**	78.61 ± 7.71	67.68 ± 1.35	52.96 ± 6.81	66.72 ± 5.36	56.06 ± 6.15	73.27 ± 1.61	85.87 ± 4.47	25.61 ± 4.35	27.49 ± 0.00	19.24 ± 3.34	36.15 ± 3.39
15	**II-15**	33.35 ± 1.75	−11.45 ± 0.26	16.72 ± 6.58	−76.28 ± 0.00	53.34 ± 4.83	27.80 ± 2.54	93.77 ± 1.45	41.82 ± 1.60	26.07 ± 5.14	50.79 ± 11.73	28.53 ± 5.47
16	**II-16**	39.16 ± 0.96	40.23 ± 7.52	18.73 ± 1.35	50.58 ± 6.99	26.94 ± 3.51	34.32 ± 4.38	64.22 ± 3.29	38.09 ± 0.23	31.44 ± 0.00	77.25 ± 3.64	26.26 ± 3.39
17	**II-17**	40.00 ± 4.73	64.36 ± 0.00	22.06 ± 0.00	65.65 ± 2.45	22.86 ± 4.78	15.17 ± 0.35	−15.52 ± 0.00	32.58 ± 2.98	32.23 ± 2.52	45.35 ± 2.43	19.03 ± 9.06
18	**I1**	25.34 ± 0.00	4.09 ± 0.00	25.56 ± 0.52	−162.93 ± 3.26	6.75 ± 0.00	19.57 ± 4.26	17.47 ± 2.37	31.28 ± 0.69	15.64 ± 5.92	36.48 ± 0.61	37.07 ± 2.71
19	**I2**	23.98 ± 1.35	−6.09 ± 0.00	16.30 ± 3.14	−155.11 ± 24.35	11.14 ± 2.58	17.78 ± 3.11	2.60 ± 0.00	32.98 ± 1.03	13.74 ± 0.00	34.19 ± 1.21	35.47 ± 1.86
20	**I3**	16.10 ± 5.18	9.14 ± 4.18	9.37 ± 0.00	11.53 ± 1.40	9.86 ± 0.00	18.27 ± 1.73	66.64 ± 0.39	7.78 ± 2.29	3.24 ± 0.00	1.65 ± 0.00	24.06 ± 6.04
21	**Eugenol**	28.92 ± 1.69	37.27 ± 8.61	47.78 ± 2.32	−167.46 ± 0.82	42.82 ± 5.11	18.02 ± 1.38	40.99 ± 1.45	9.32 ± 2.64	43.44 ± 0.00	22.82 ± 3.54	13.93 ± 0.00
22	**Paoneol**	31.71 ± 2.70	82.14 ± 0.96	8.57 ± 0.00	−168.53 ± 4.66	16.69 ± 7.80	55.83 ± 1.15	65.15 ± 12.49	14.26 ± 0.69	9.56 ± 0.00	8.30 ± 1.62	28.81 ± 0.00
23	**Syringol**	9.80 ± 3.38	−7.95 ± 0.32	−2.70 ± 0.37	−172.65 ± 0.00	−15.68 ± 1.76	8.57 ± 0.00	6.69 ± 1.05	7.21 ± 0.34	9.32 ± 4.32	0.00 ± 1.62	−6.98 ± 0.00
24	**Azoxystrobin**	86.73 ± 0.17	54.32 ± 0.45	58.89 ± 3.07	31.80 ± 7.22	63.51 ± 2.09	71.32 ± 1.38	96.84 ± 0.00	51.46 ± 0.11	60.19 ± 17.20	83.83 ± 1.42	47.69 ± 14.45

**Table 3:** The EC_50_ values of the selective compounds against several strains of fungi.

Compd. No.	EC_50_ (mg/L)				
	*Pestalotiopsis theae*	*Phomopsis adianticola*	*Gibberella zeae*	*Sclerotinia sclerotiorum*	*Magnapothe grisea*
II-1	-	-	-	6.75 ± 0.12	-
II-2	0.25 ± 0.02	-	-	0.64 ± 0.06	0.96 ± 0.08
II-3	-	-	-	0.1 ± 0.01	2.05 ± 0.13
II-5	-	-	-	-	-
II-7	-	-	-	0.25 ± 0.05	-
II-8	0.04 ± 0.01	3.06 ± 0.29	-	0.003 ± 0.00	0.95 ± 0.08
II-9	-	-	-	0.79 ± 0.06	-
II-13	-	-	15.48 ± 1.76	0.1 ± 0.02	-
Azoxystrobin	2.54 ± 0.38	35.3 ± 3.65	36.69 ± 3.69	18.75 ± 1.10	2.41 ± 0.30

## Supplementary Materials

Supplementary materials are available online.
