# Supplementary material for: Synthesis and Evaluation of Essential Oil-Derived β-Methoxyacrylate Derivatives as High Potential Fungicides
Source: Molecules. 2017 May 8;22(5):763. doi: 10.3390/molecules22050763 (PMC6154096; doi:10.3390/molecules22050763)
Supplement: Supplementary file 1 [file molecules-22-00763-s001.pdf]

Synthesis and evaluation of essential oils-oriented β-methoxyacrylate derivatives as highly potential fungicides

Haihuan Su, Wenda Wang, Longzhu Bao, Shuangshuang Wang, Xiufang Cao*

College of Science, Huazhong Agricultural University, Wuhan, 430070, China
*Corresponding author: caoxiufang@mail.hzau.edu.cn

Supporting Information

Representative ^{1}H-NMR, and ^{13}C-NMR spectra for the synthesized compounds

Compd. II-1

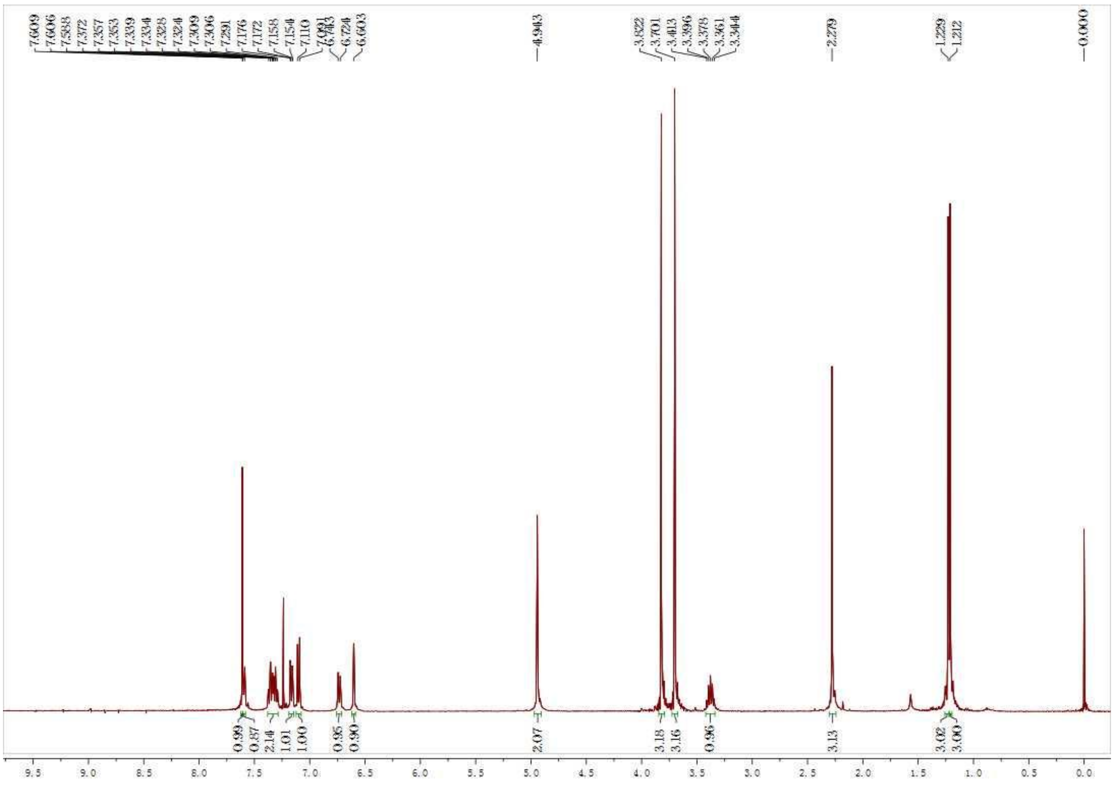

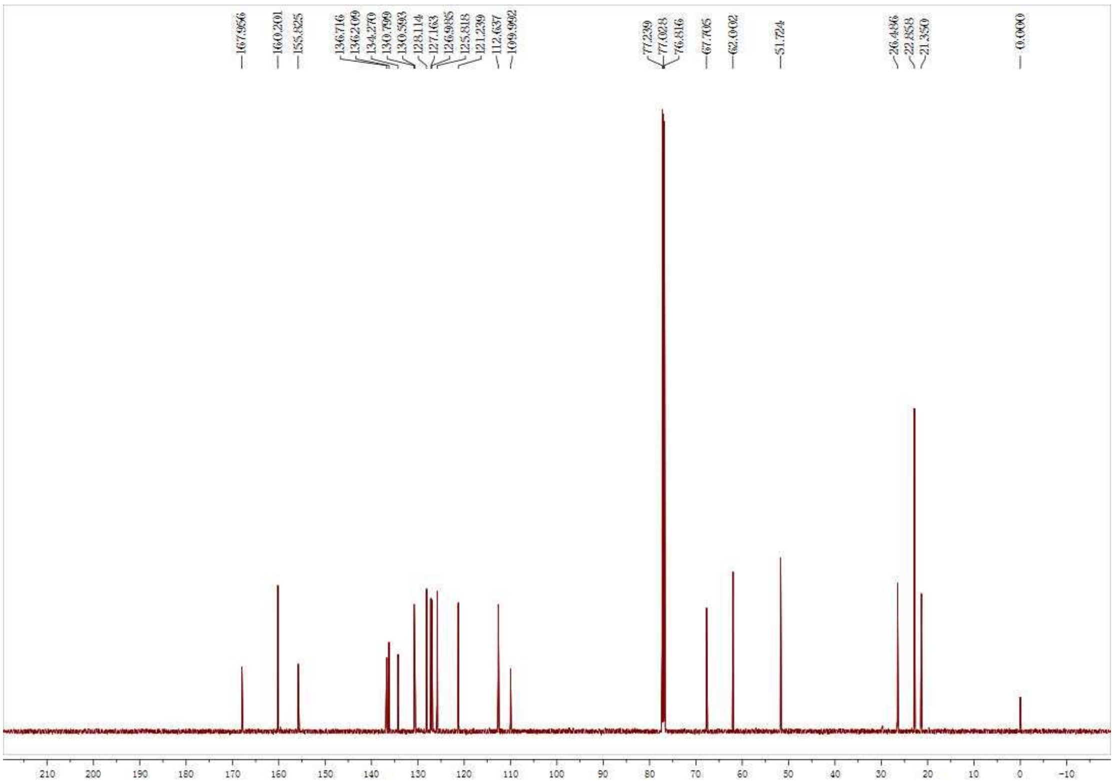
167.956
160.201
155.825
136.716
136.209
134.270
130.799
130.593
128.114
127.163
126.985
125.818
121.239
112.637
109.982
77.239
77.028
76.816
67.705
62.002
51.724
26.436
22.858
21.350
0.000

Compd. II-2

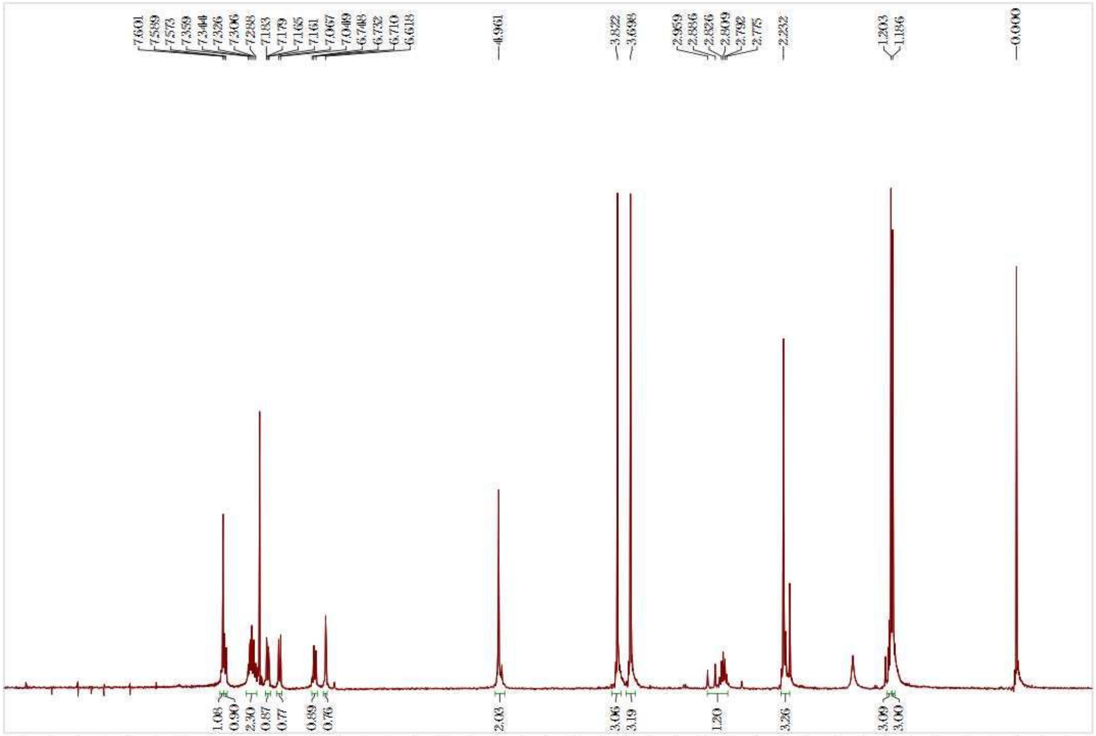
7.601
7.589
7.573
7.359
7.344
7.326
7.306
7.288
7.183
7.179
7.165
7.161
7.067
7.049
6.748
6.732
6.710
6.618
4.961
3.822
3.698
2.959
2.836
2.826
2.809
2.792
2.775
2.232
1.203
1.186
0.000
1.08
0.90
2.30
0.87
0.77
0.89
0.76
2.03
3.06
3.19
1.20
3.26
3.09
3.00

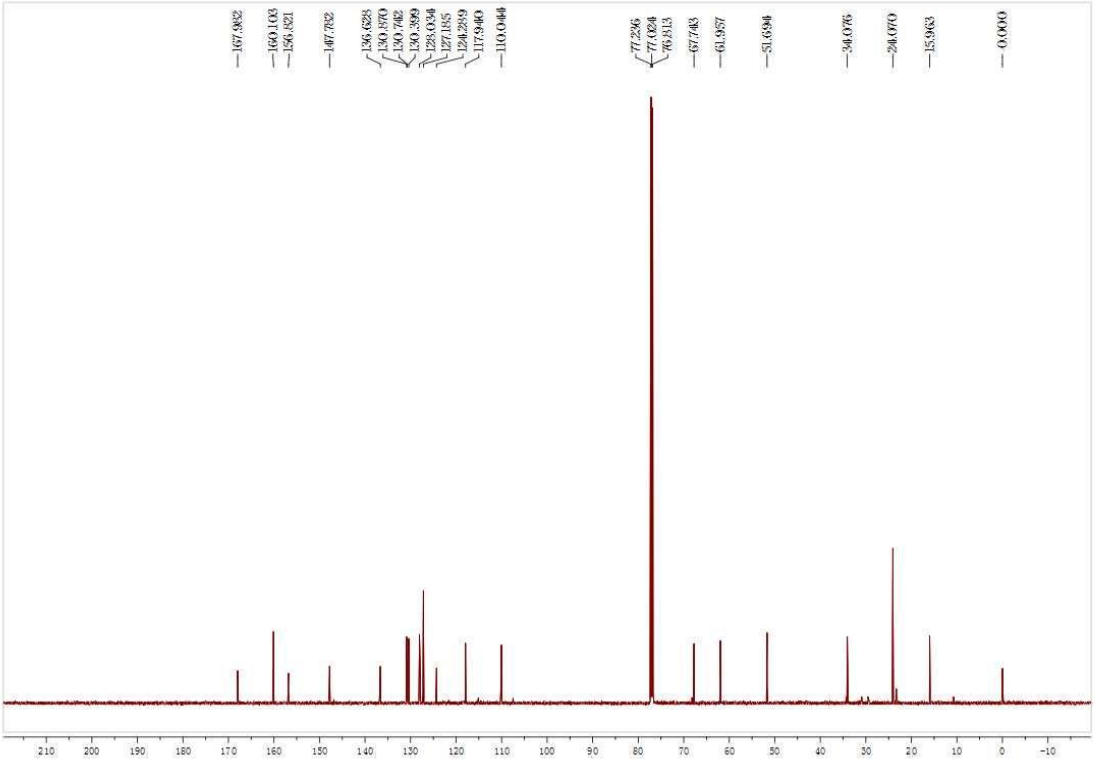

Compd. II-3

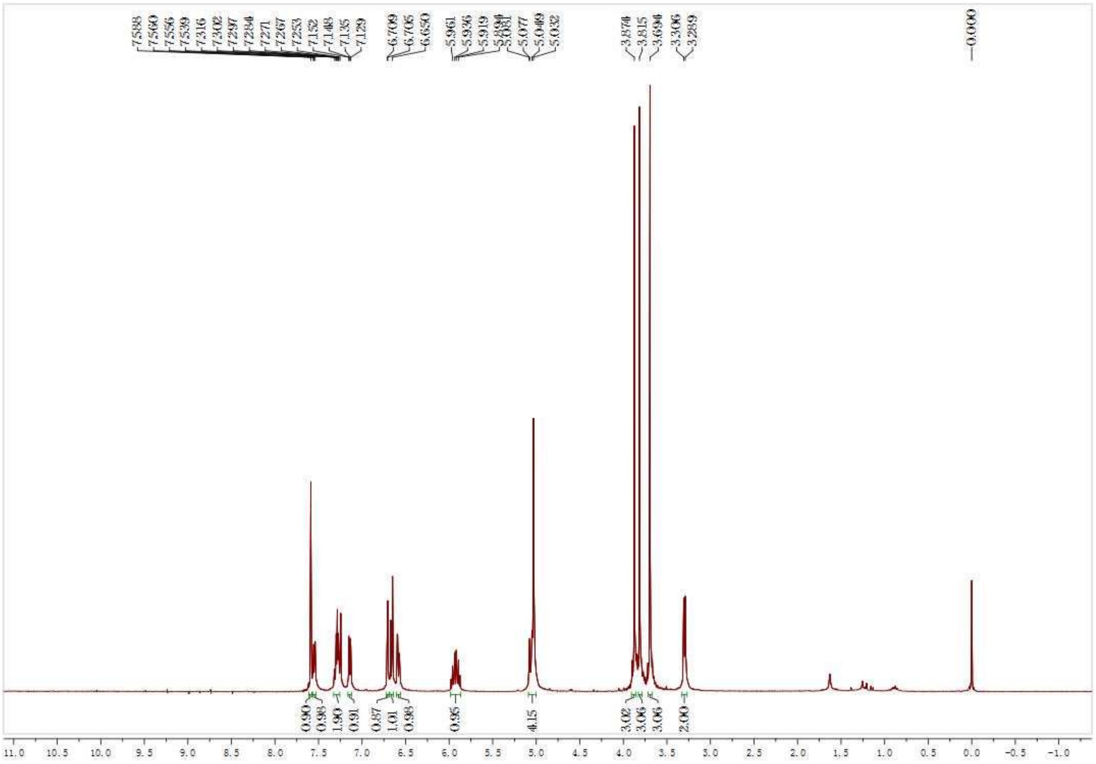

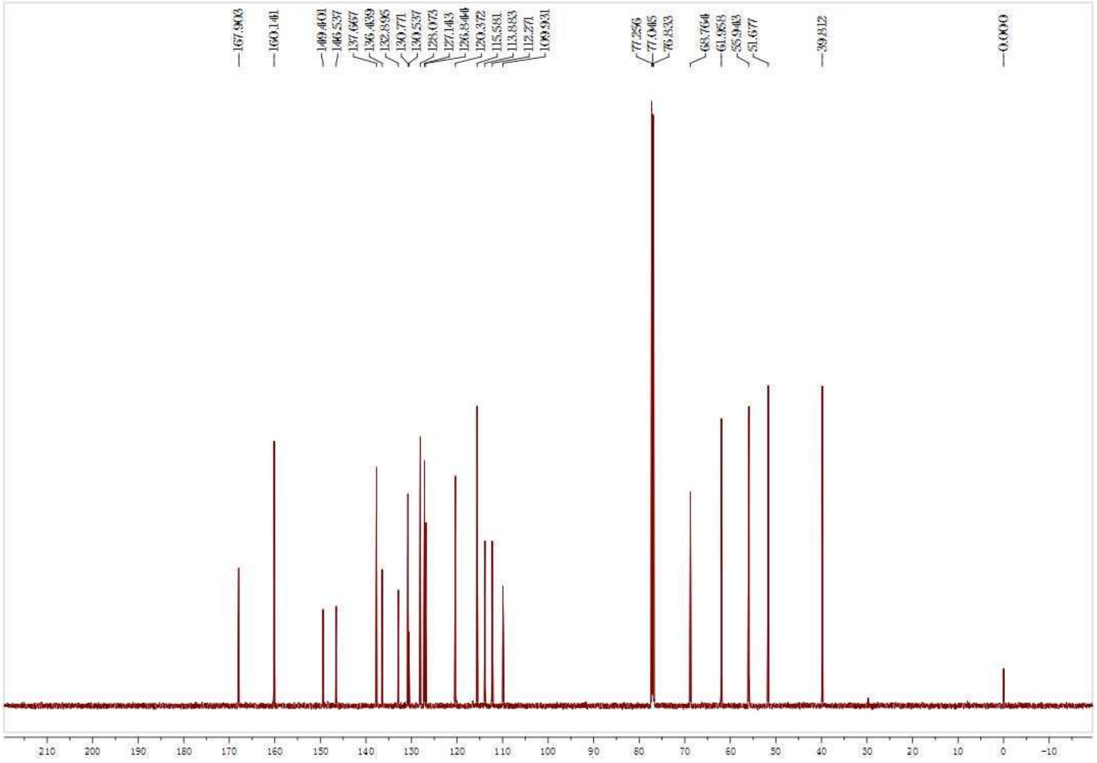
167.903
160.141
149.401
146.537
137.667
136.439
132.895
130.771
130.537
128.073
127.143
126.844
120.372
115.581
113.883
112.271
109.931
77.256
77.045
76.833
68.764
61.958
55.943
51.677
39.812
0.000

Compd. II-4

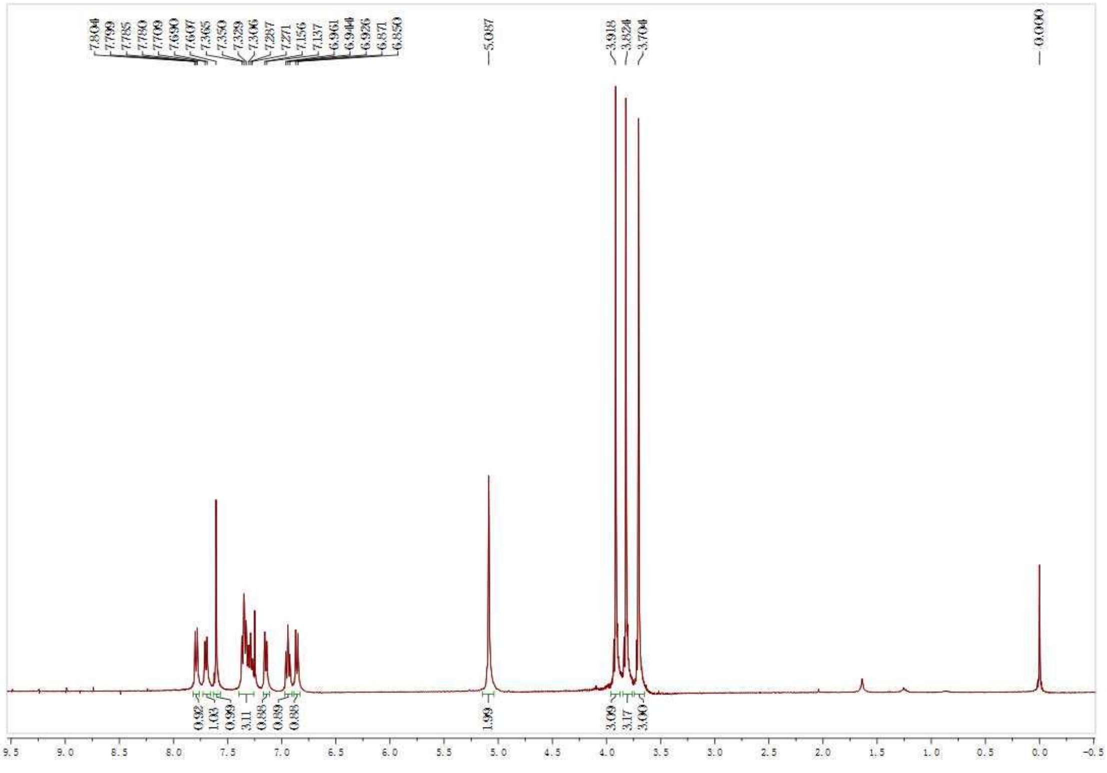
7.804
7.799
7.785
7.780
7.709
7.690
7.607
7.365
7.350
7.329
7.306
7.287
7.271
7.156
7.137
6.961
6.944
6.926
6.871
6.850
5.087
3.918
3.824
3.704
0.000
0.92
1.03
0.99
3.11
0.88
0.89
0.88
1.99
3.09
3.17
3.00

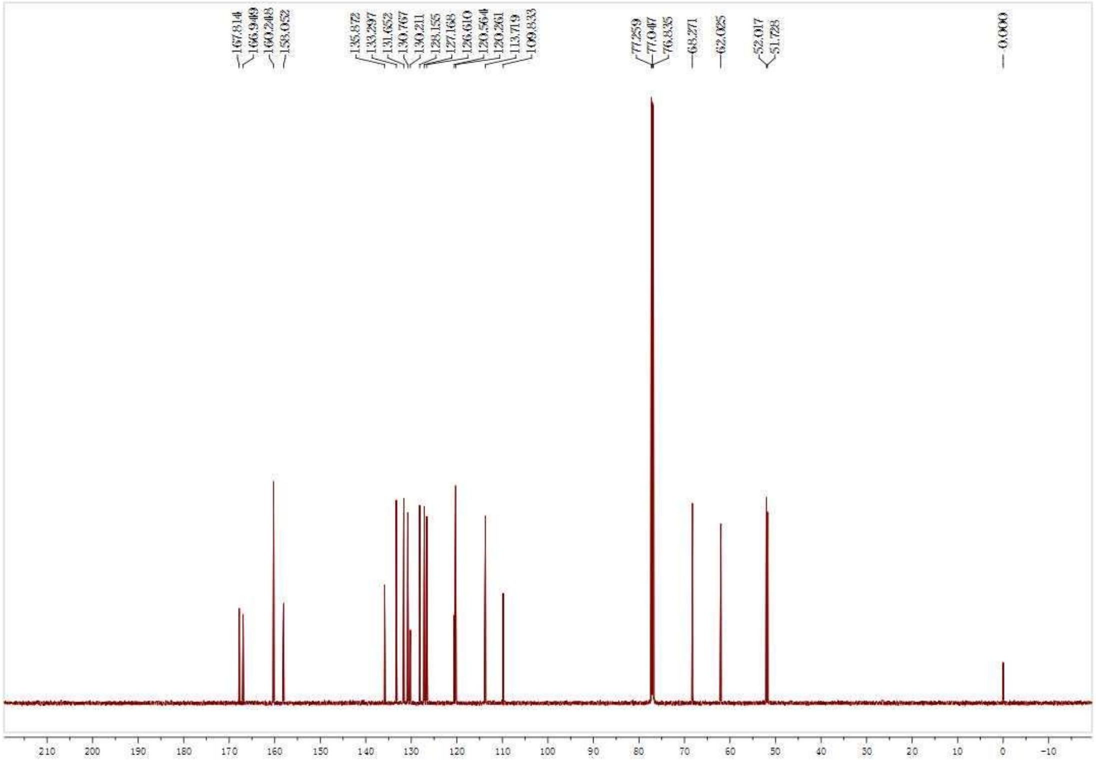
167.814
166.949
160.248
158.052
135.872
133.297
131.652
130.767
130.211
128.155
127.168
126.610
120.564
120.261
113.719
109.833
77.259
77.047
76.835
68.271
62.025
52.017
51.728
0.000
210 200 190 180 170 160 150 140 130 120 110 100 90 80 70 60 50 40 30 20 10 0 -10

Compd. II-5

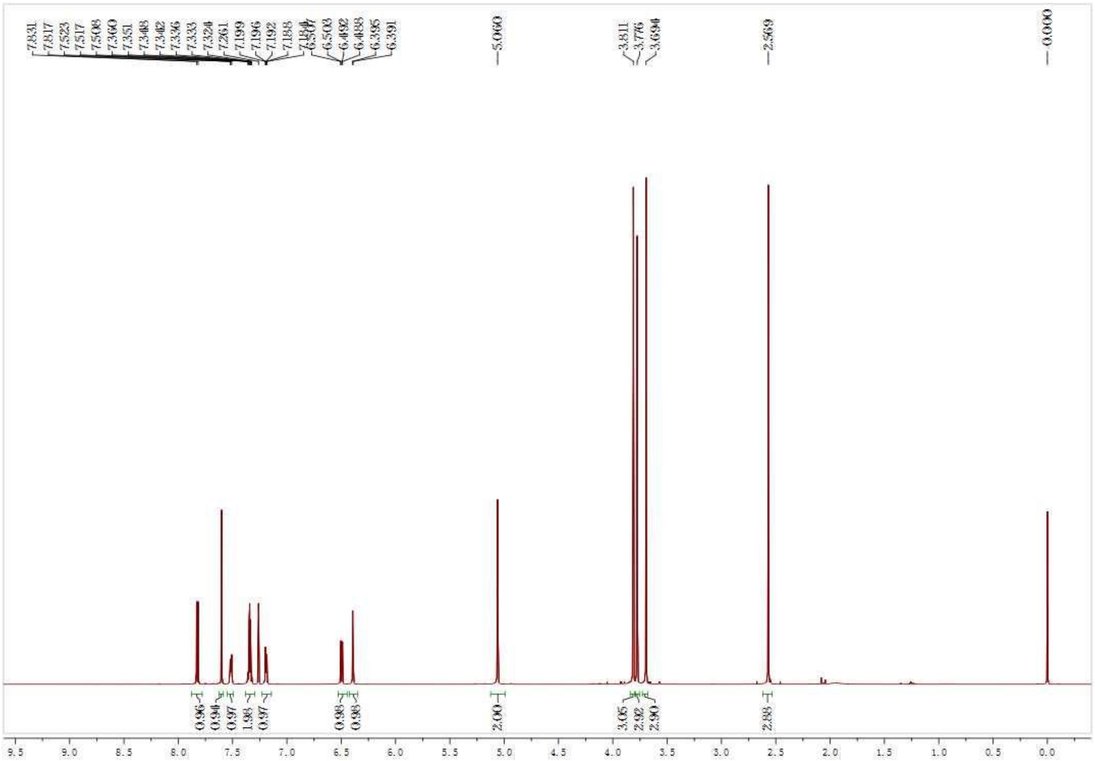
7.831
7.817
7.523
7.517
7.508
7.360
7.351
7.348
7.342
7.336
7.333
7.324
7.261
7.199
7.196
7.192
7.188
6.503
6.492
6.488
6.395
6.391
5.060
3.811
3.776
3.694
2.569
0.000
0.96
0.94
0.97
1.98
0.97
0.98
0.98
2.00
3.05
2.92
2.90
2.88
9.5 9.0 8.5 8.0 7.5 7.0 6.5 6.0 5.5 5.0 4.5 4.0 3.5 3.0 2.5 2.0 1.5 1.0 0.5 0.0

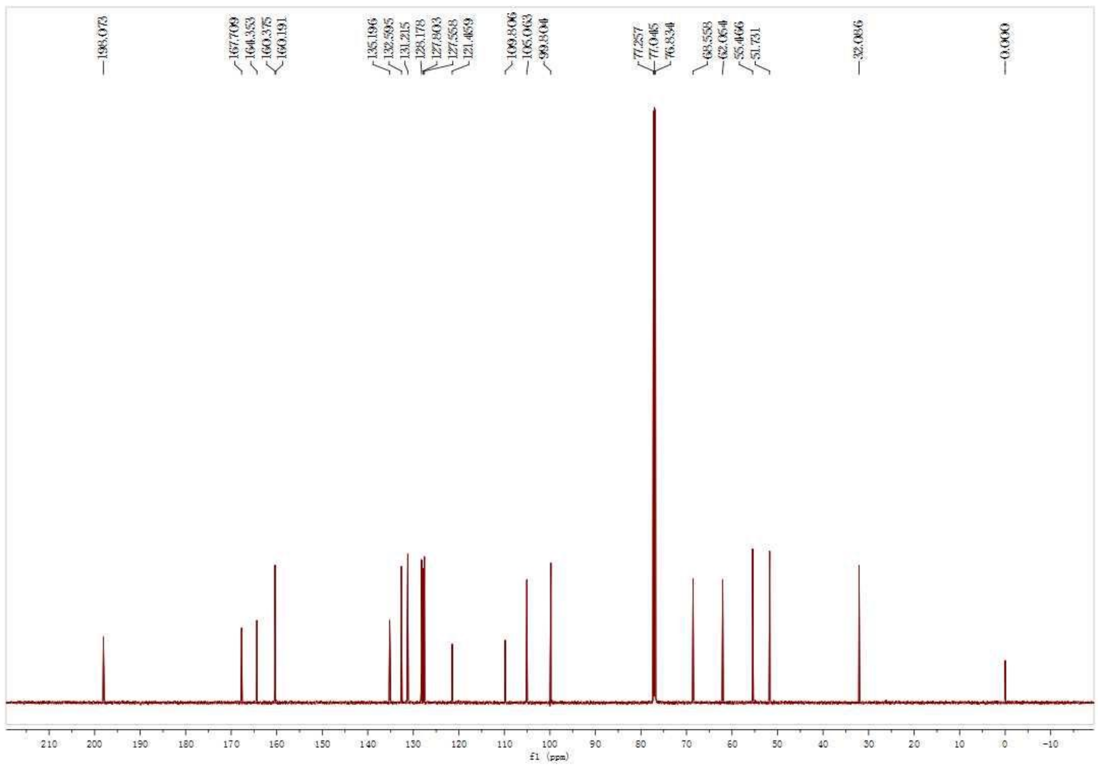

Compd. II-6

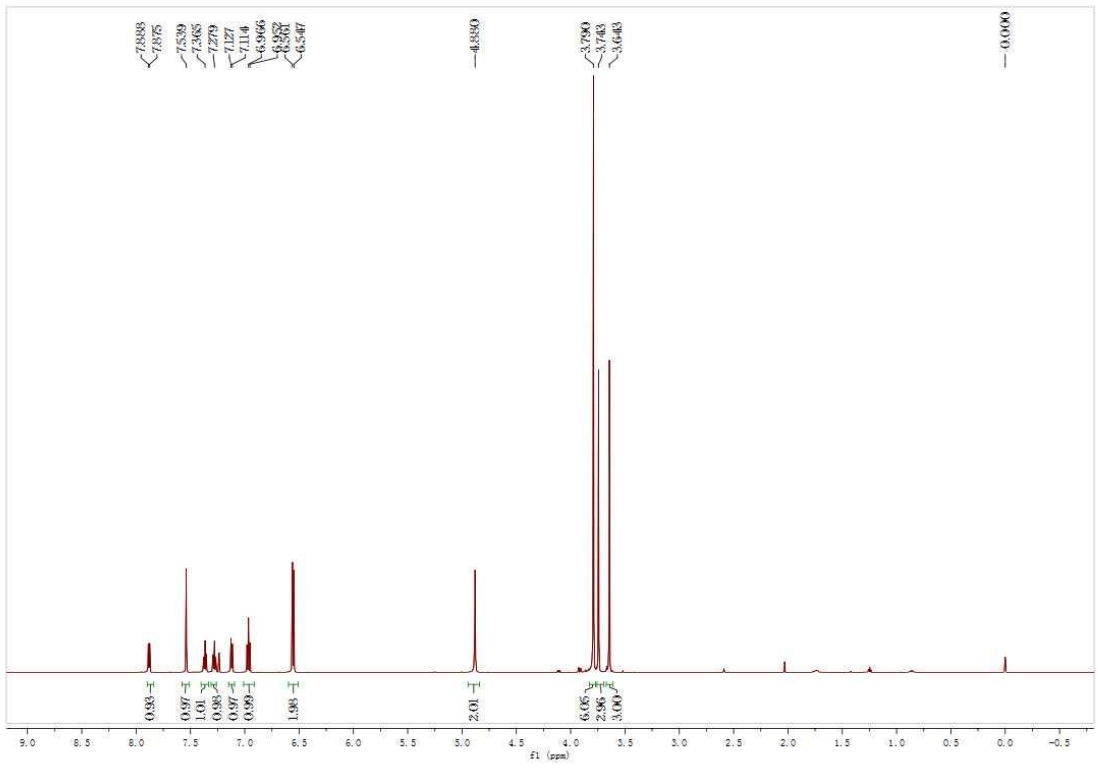

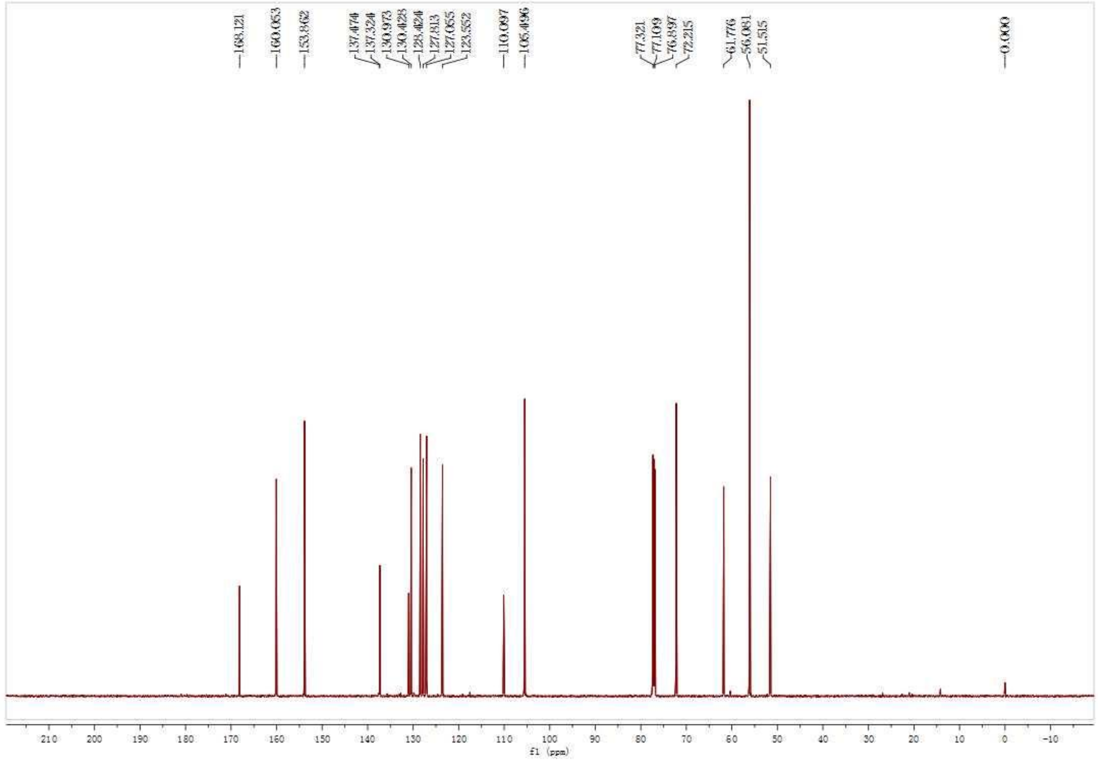

168.121
160.053
153.862
137.474
137.324
130.973
130.423
128.424
127.813
127.055
123.552
110.097
105.496
77.321
77.109
76.897
72.215
61.776
56.081
51.515
0.000
f1 (ppm)

Compd. II-7

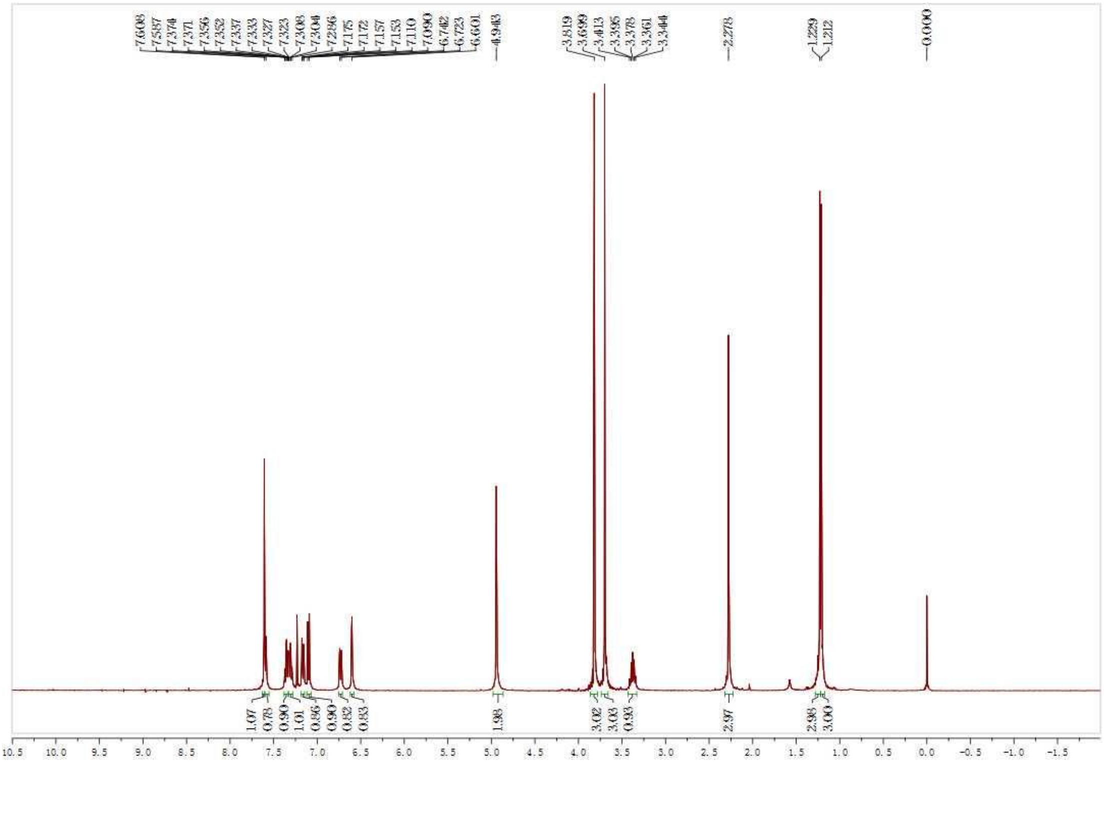

7.608
7.587
7.374
7.371
7.356
7.352
7.337
7.333
7.327
7.323
7.308
7.304
7.286
7.175
7.172
7.157
7.153
7.110
7.090
6.742
6.723
6.601
4.943
3.819
3.699
3.413
3.395
3.378
3.361
3.344
2.273
1.229
1.212
0.000
1.07
0.78
0.90
1.01
0.86
0.90
0.82
0.83
1.98
3.02
3.03
0.93
2.97
2.98
3.00

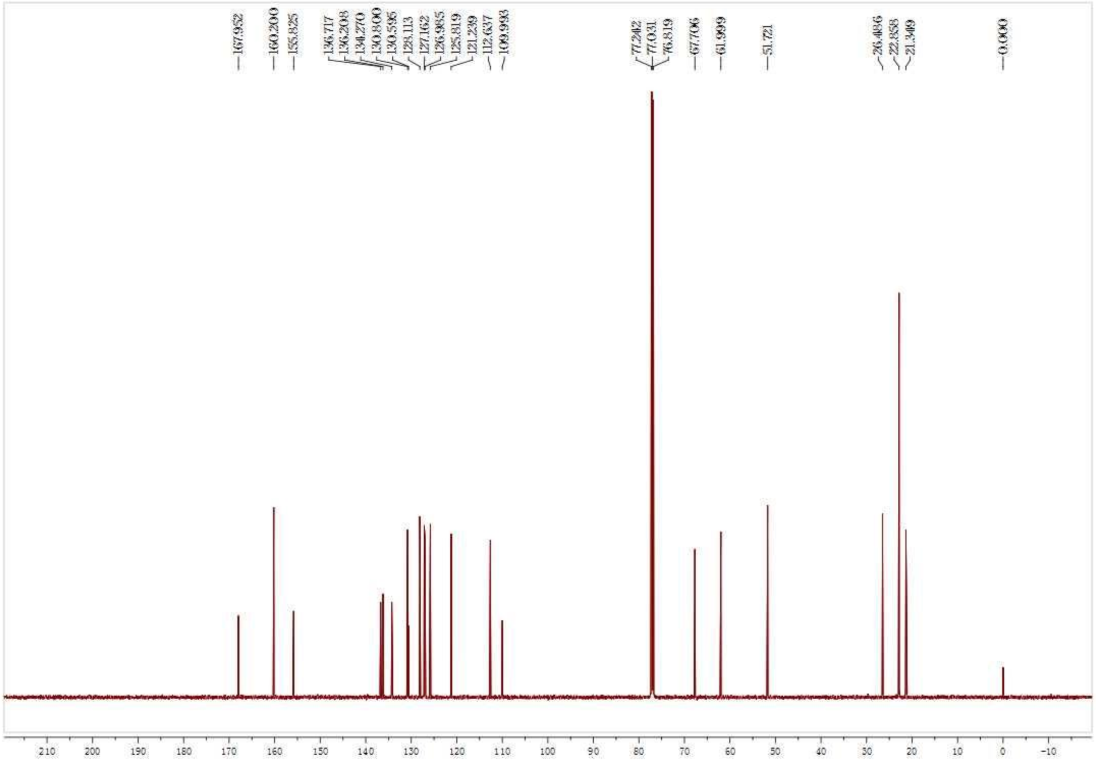
167.952
160.200
155.825
136.717
136.208
134.270
130.800
130.595
128.113
127.162
126.985
125.819
121.239
112.637
109.993
77.242
77.031
76.819
67.706
61.999
51.721
26.486
22.858
21.349
0.000

Compd. II-8

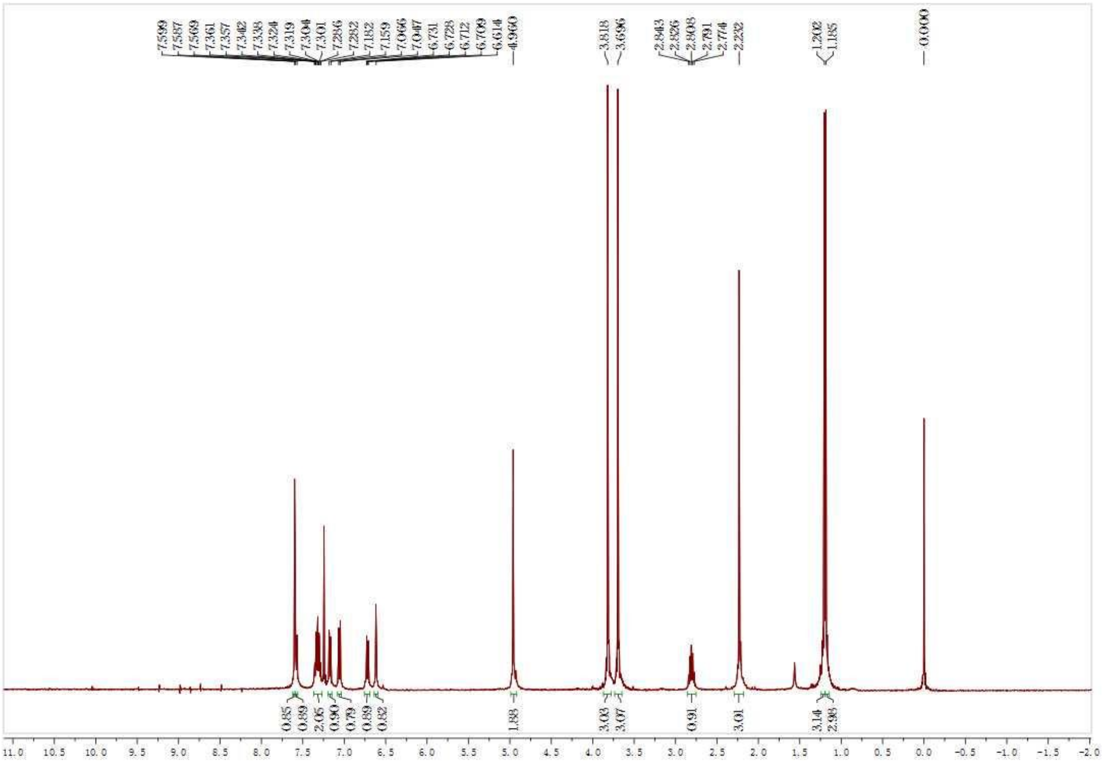
7.599
7.587
7.569
7.361
7.357
7.342
7.338
7.324
7.319
7.304
7.301
7.286
7.282
7.182
7.159
7.066
7.047
6.731
6.728
6.712
6.709
6.614
4.960
3.818
3.696
2.843
2.826
2.808
2.791
2.774
2.232
1.202
1.185
0.000
0.85
0.89
2.05
0.90
0.79
0.89
0.82
1.88
3.03
3.07
0.91
3.01
3.14
2.98

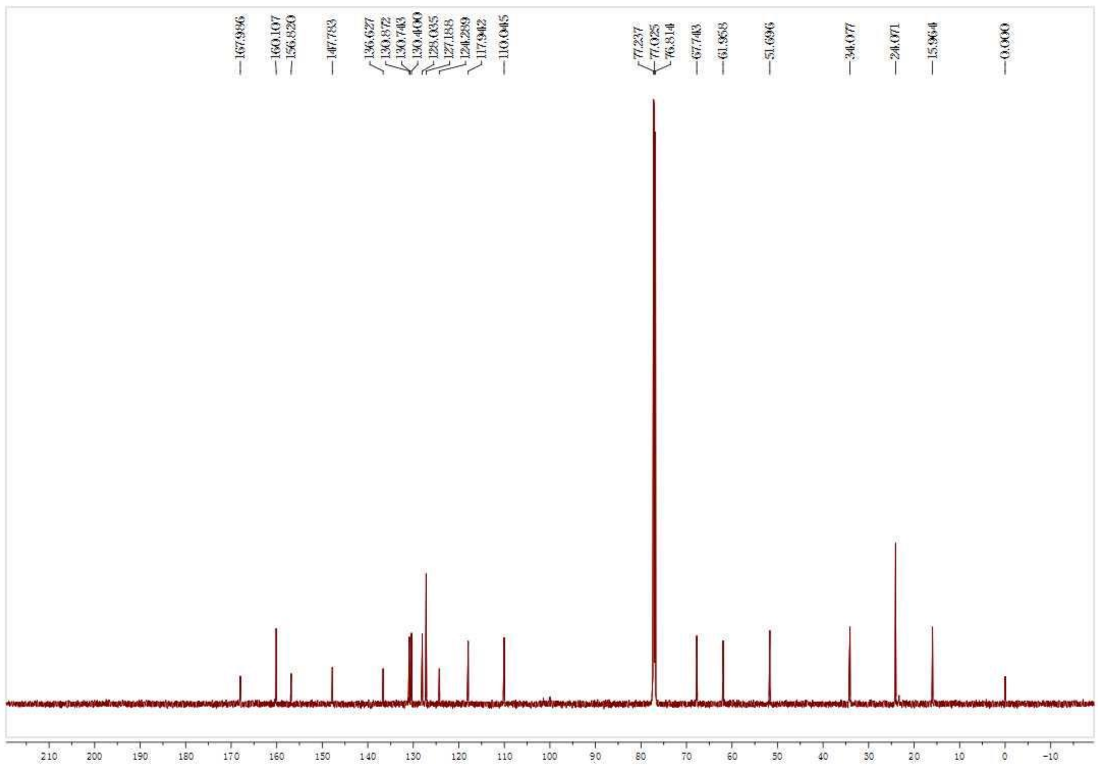

167.936
160.107
156.820
147.783
136.627
130.872
130.743
130.400
128.035
127.188
124.289
117.942
110.045
77.237
77.025
76.814
67.743
61.958
51.696
34.077
24.071
15.964
0.000
210 200 190 180 170 160 150 140 130 120 110 100 90 80 70 60 50 40 30 20 10 0 -10

Compd. II-9

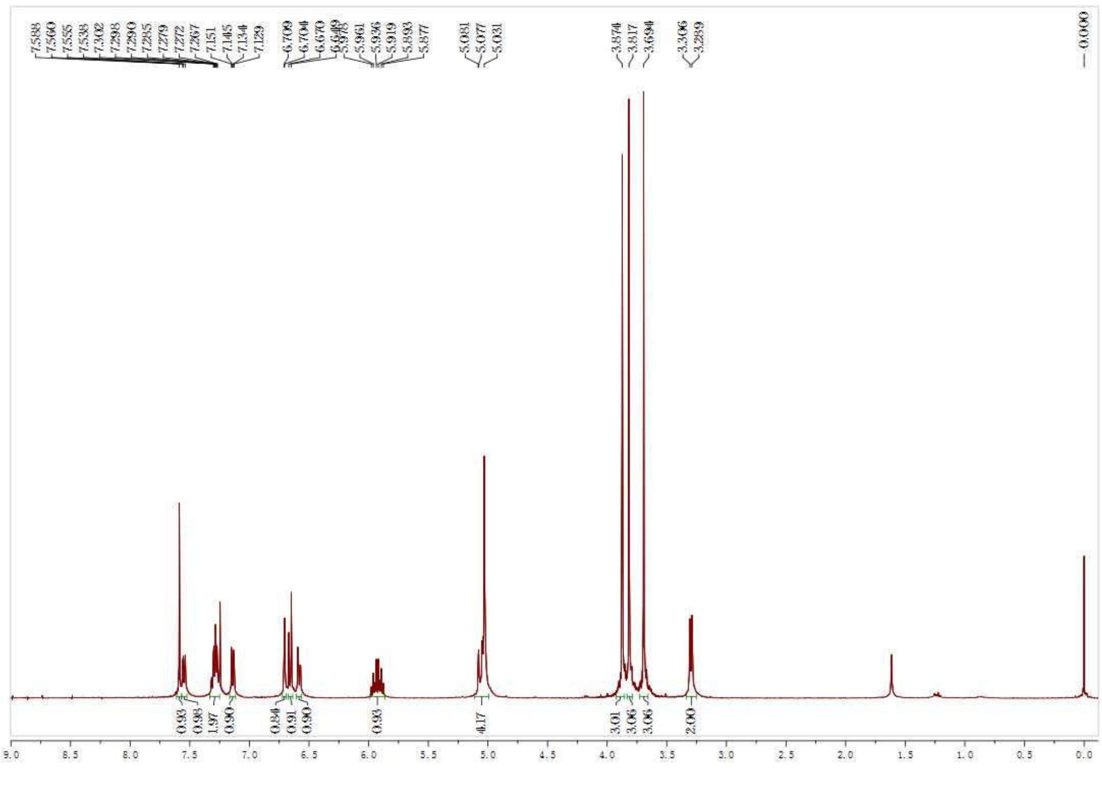

7.588
7.560
7.555
7.538
7.302
7.298
7.290
7.285
7.279
7.272
7.267
7.151
7.145
7.134
7.129
6.709
6.704
6.670
5.961
5.936
5.919
5.893
5.877
5.081
5.077
5.031
3.874
3.817
3.694
3.306
3.289
0.000
0.93
0.98
1.97
0.90
0.84
0.91
0.90
0.93
4.17
3.01
3.06
3.06
2.00
9.0 8.5 8.0 7.5 7.0 6.5 6.0 5.5 5.0 4.5 4.0 3.5 3.0 2.5 2.0 1.5 1.0 0.5 0.0

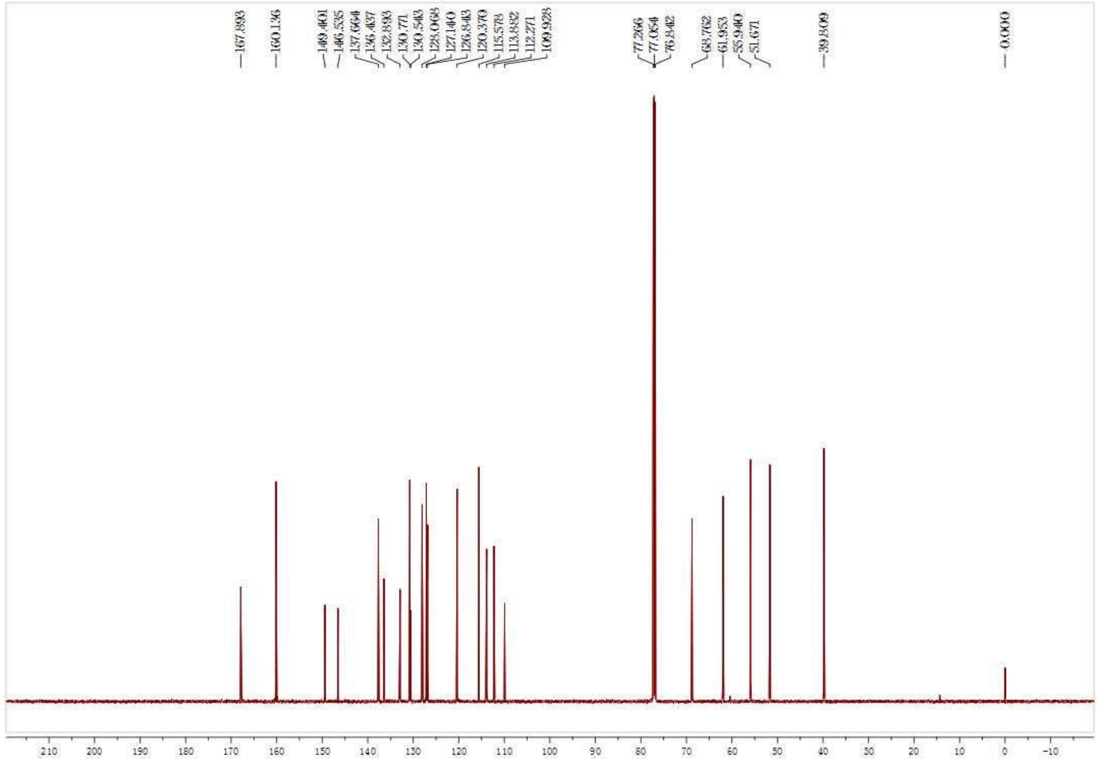
167.893
160.136
149.401
146.535
137.664
136.437
132.893
130.771
130.543
128.068
127.140
126.843
120.370
115.578
113.882
112.271
109.928
77.266
77.054
76.842
68.762
61.953
55.940
51.671
39.809
0.000

Compd. II-10

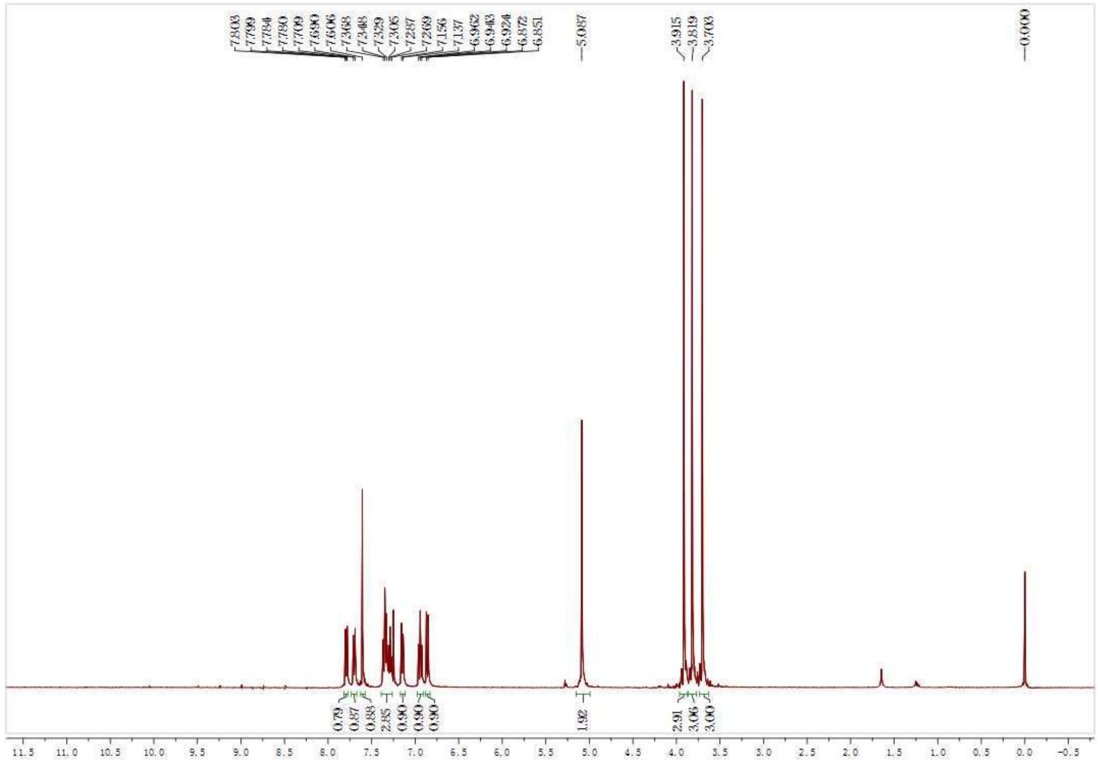
7.803
7.799
7.784
7.780
7.709
7.690
7.606
7.368
7.348
7.329
7.305
7.287
7.269
7.156
7.137
6.962
6.943
6.924
6.872
6.851
5.087
3.915
3.819
3.703
0.000
0.79
0.87
0.88
2.85
0.90
0.90
0.90
1.92
2.91
3.06
3.00

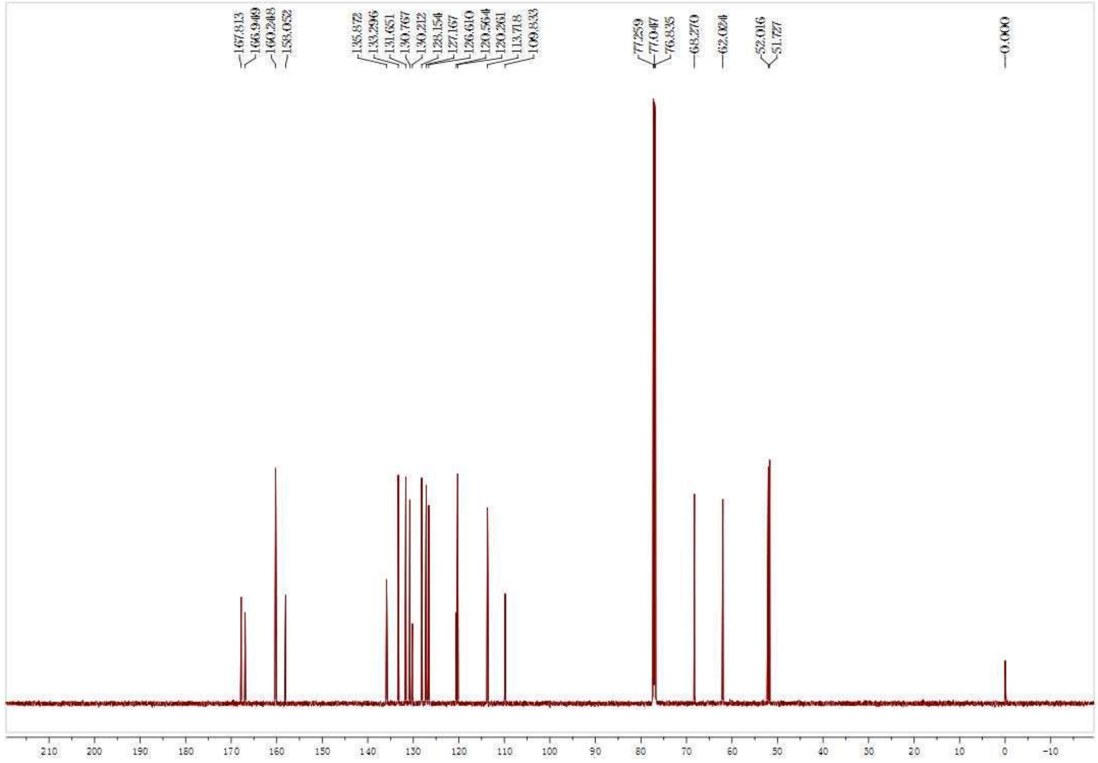
167.813
166.949
160.248
158.052
135.872
133.296
131.651
130.767
130.212
128.154
127.167
126.610
120.564
120.261
113.718
109.833
77.259
77.047
76.835
68.270
62.024
52.016
51.727
0.000

Compd. II-11

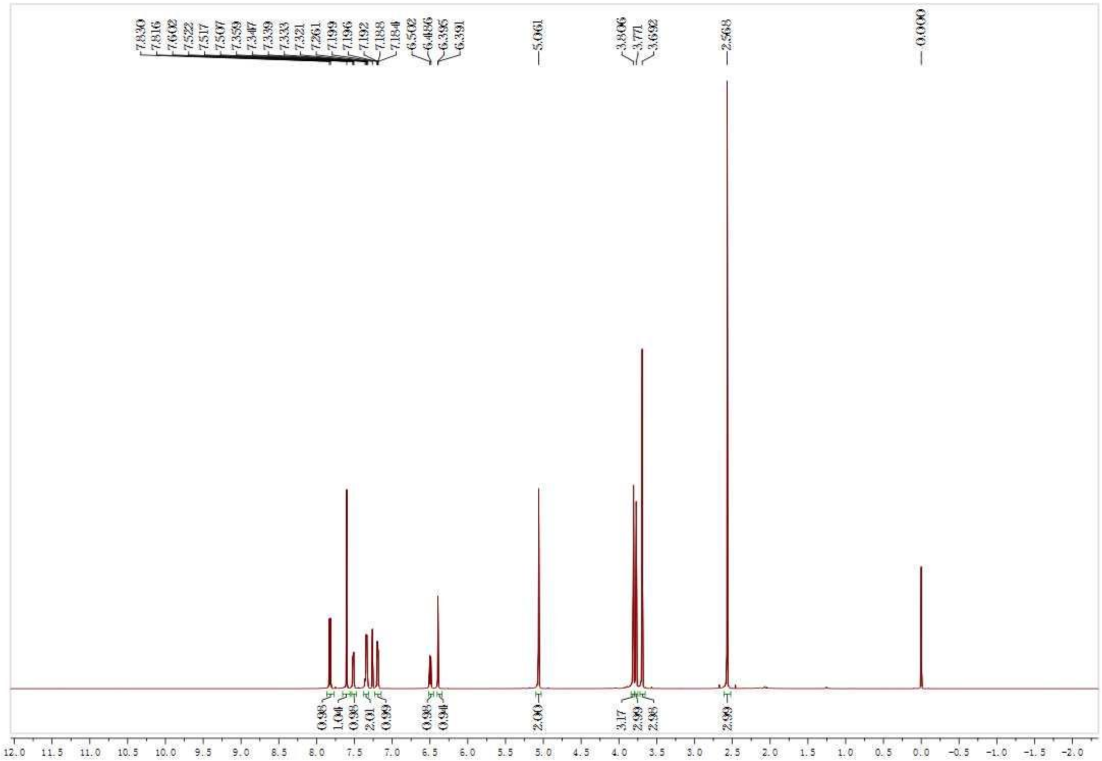
7.830
7.816
7.602
7.522
7.517
7.507
7.359
7.347
7.339
7.333
7.321
7.261
7.199
7.196
7.192
7.188
7.184
6.502
6.486
6.395
6.391
5.061
3.806
3.771
3.692
2.568
0.000

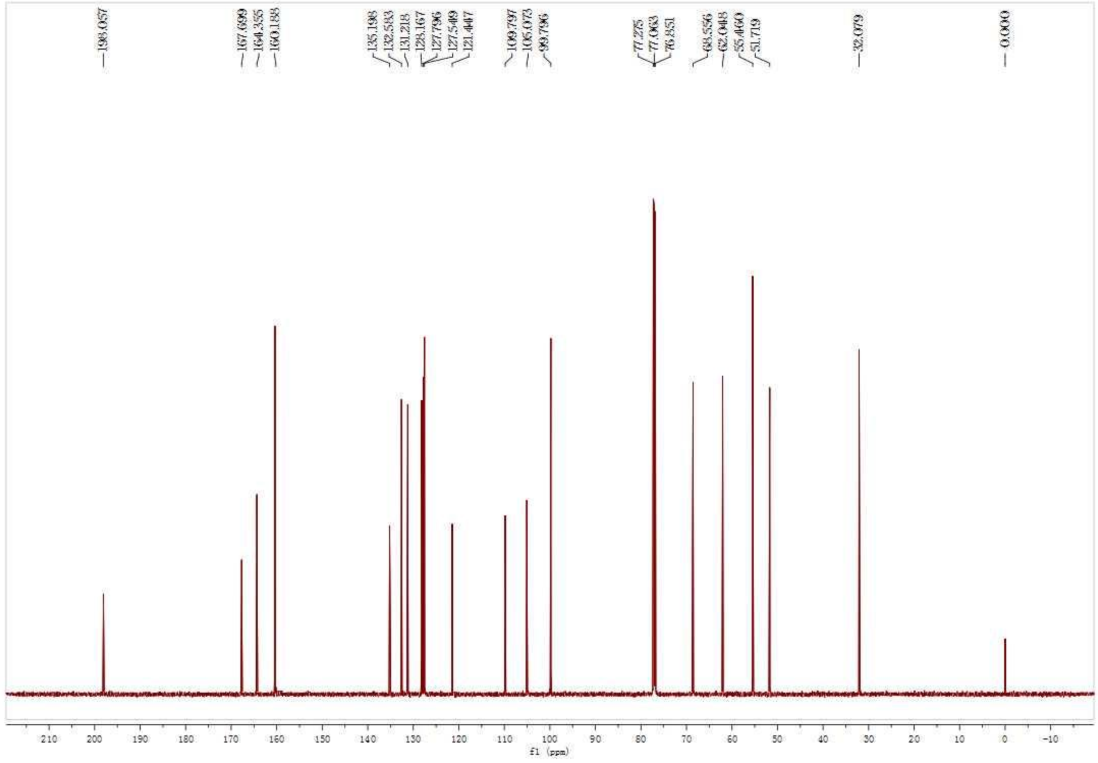

Compd. II-12

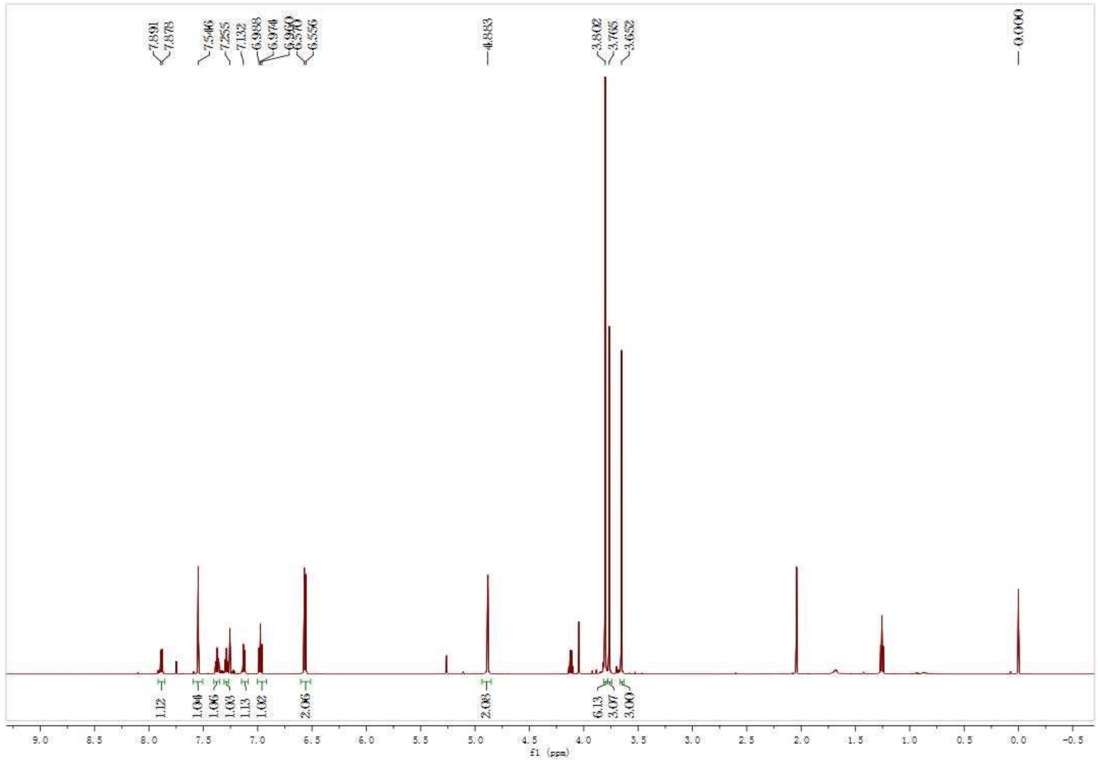

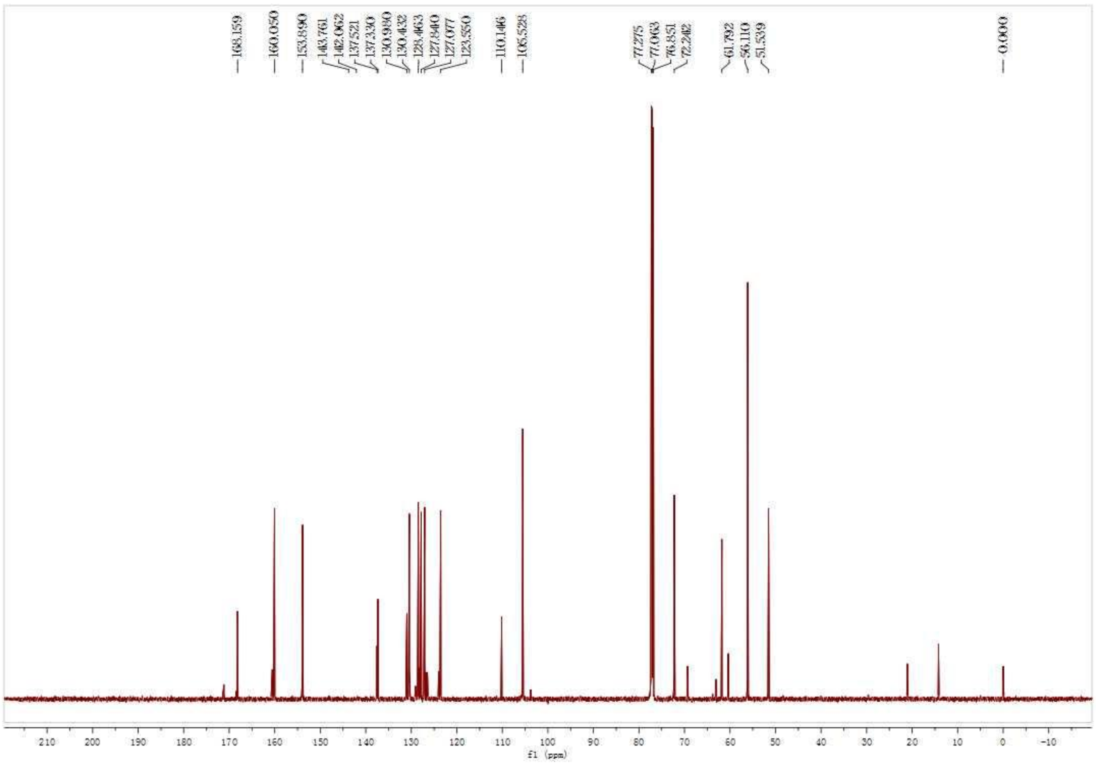
168.159
160.050
153.890
143.761
142.062
137.521
137.330
130.980
130.432
128.463
127.840
127.077
123.550
110.146
105.528
77.275
77.063
76.851
72.242
61.792
56.110
51.539
0.000
f1 (ppm)

Compd. II-13

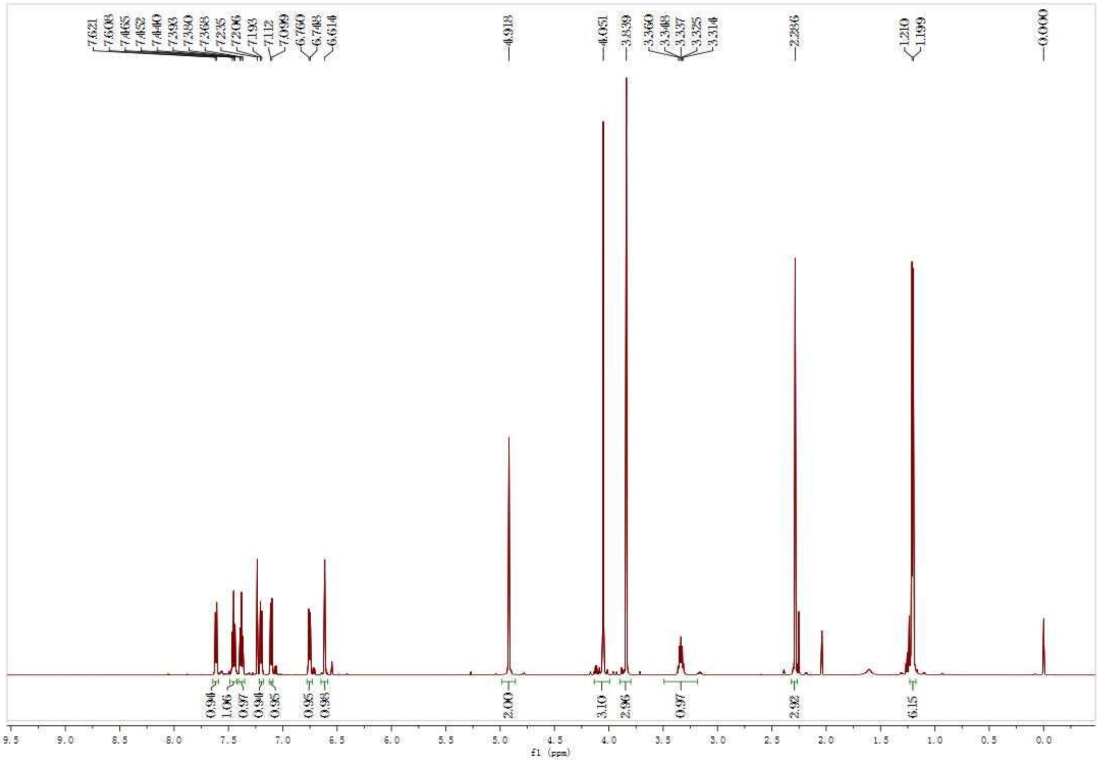
7.621
7.608
7.465
7.452
7.440
7.393
7.380
7.368
7.235
7.206
7.193
7.112
7.099
6.760
6.748
6.614
4.918
4.051
3.839
3.360
3.348
3.337
3.325
3.314
2.286
1.210
1.199
0.000
0.94
1.06
0.97
0.94
0.95
0.95
0.98
2.00
3.10
2.96
0.97
2.92
6.15
f1 (ppm)

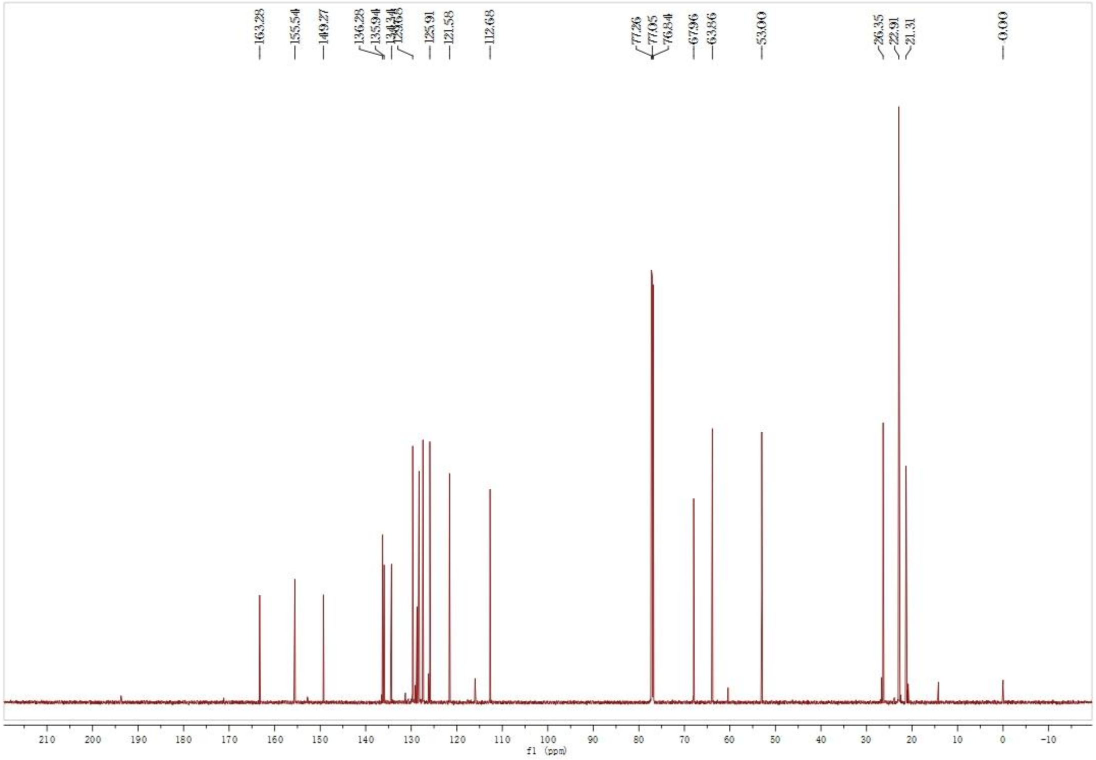

163.23
155.54
149.27
136.23
135.94
125.91
121.58
112.68
77.26
77.05
76.84
67.96
63.86
53.00
26.35
22.91
21.31
0.00
f1 (ppm)

Compd. II-14

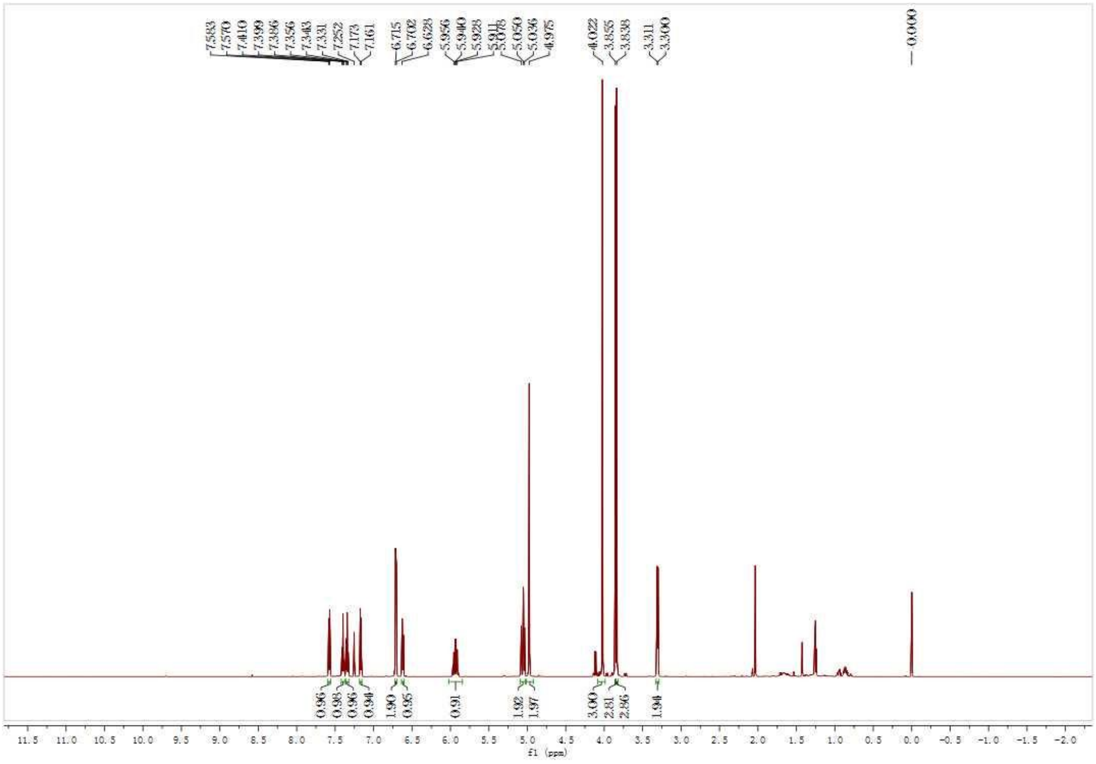

7.583
7.570
7.410
7.399
7.386
7.356
7.343
7.331
7.252
7.173
7.161
6.715
6.702
6.629
5.956
5.940
5.928
5.911
5.078
5.050
5.036
4.975
4.022
3.855
3.838
3.311
3.300
0.000
f1 (ppm)

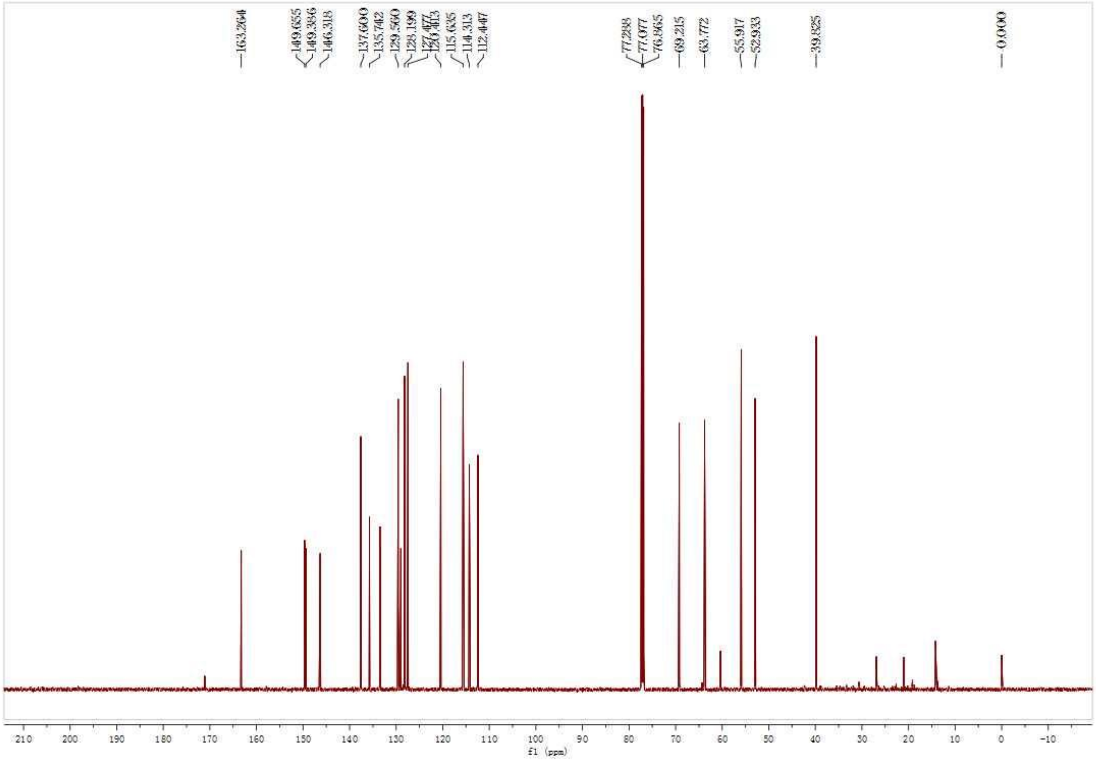
163.264
149.655
149.386
146.318
137.600
135.742
129.560
128.199
115.635
114.313
112.447
77.288
77.077
76.865
69.215
63.772
55.917
52.933
39.825
0.000
f1 (ppm)

Compd. II-15

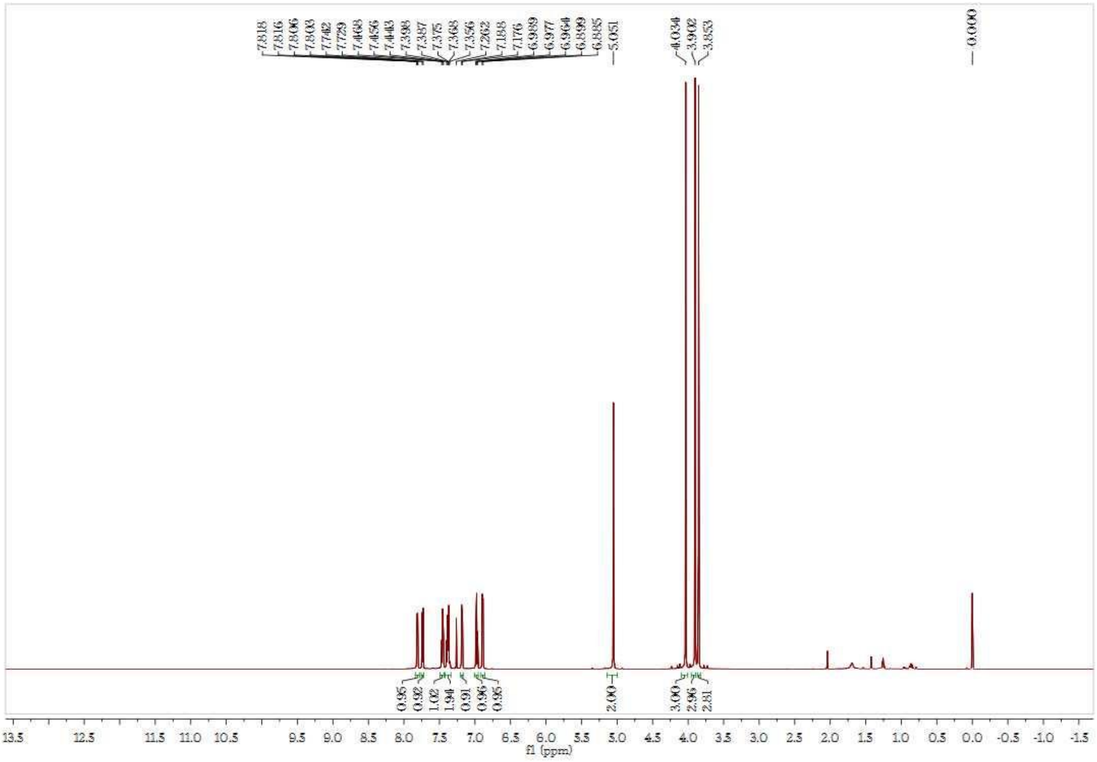
7.818
7.816
7.806
7.803
7.742
7.729
7.468
7.456
7.443
7.398
7.387
7.375
7.368
7.356
7.262
7.188
7.176
6.989
6.977
6.964
6.899
6.885
5.051
4.034
3.902
3.853
0.000
0.95
0.92
1.02
1.94
0.91
0.96
0.95
2.00
3.00
2.96
2.81
f1 (ppm)

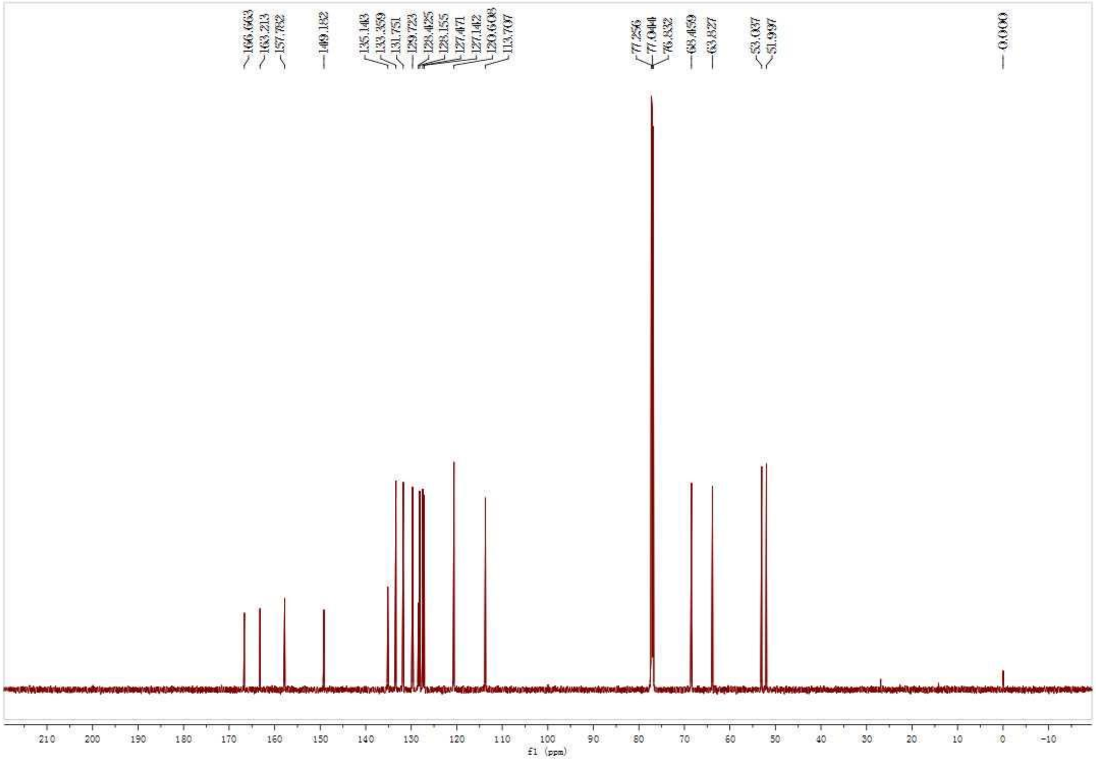
166.663
163.213
157.782
149.182
135.143
133.359
131.751
129.723
128.425
128.155
127.471
127.142
120.608
113.707
77.256
77.044
76.832
68.459
63.827
53.037
51.997
0.000
f1 (ppm)

Compd. II-16

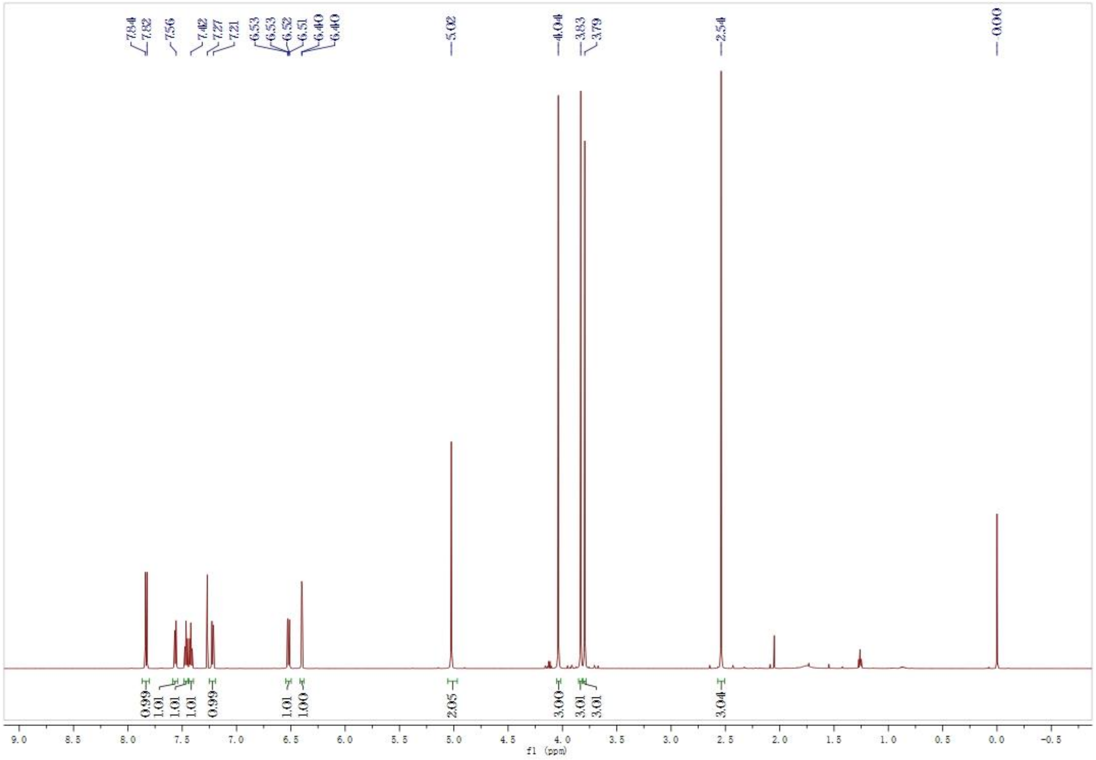
7.84
7.82
7.56
7.42
7.27
7.21
6.53
6.53
6.52
6.51
6.40
6.40
5.02
4.04
3.83
3.79
2.54
0.00
0.99
1.01
1.01
1.01
0.99
1.01
1.00
2.05
3.00
3.01
3.01
3.04
f1 (ppm)

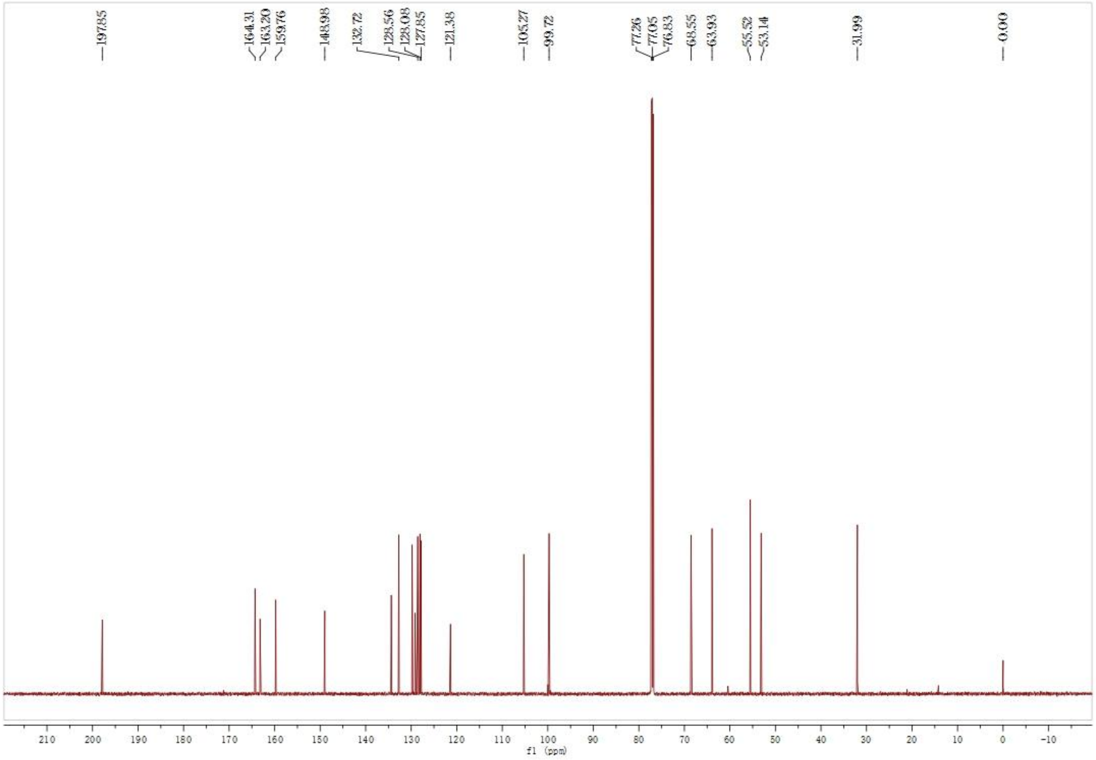
197.85
164.31
163.20
159.76
148.93
132.72
128.56
128.08
127.85
121.38
105.27
99.72
77.26
77.05
76.83
68.55
63.93
55.52
53.14
31.99
0.00
f1 (ppm)

Compd. II-17

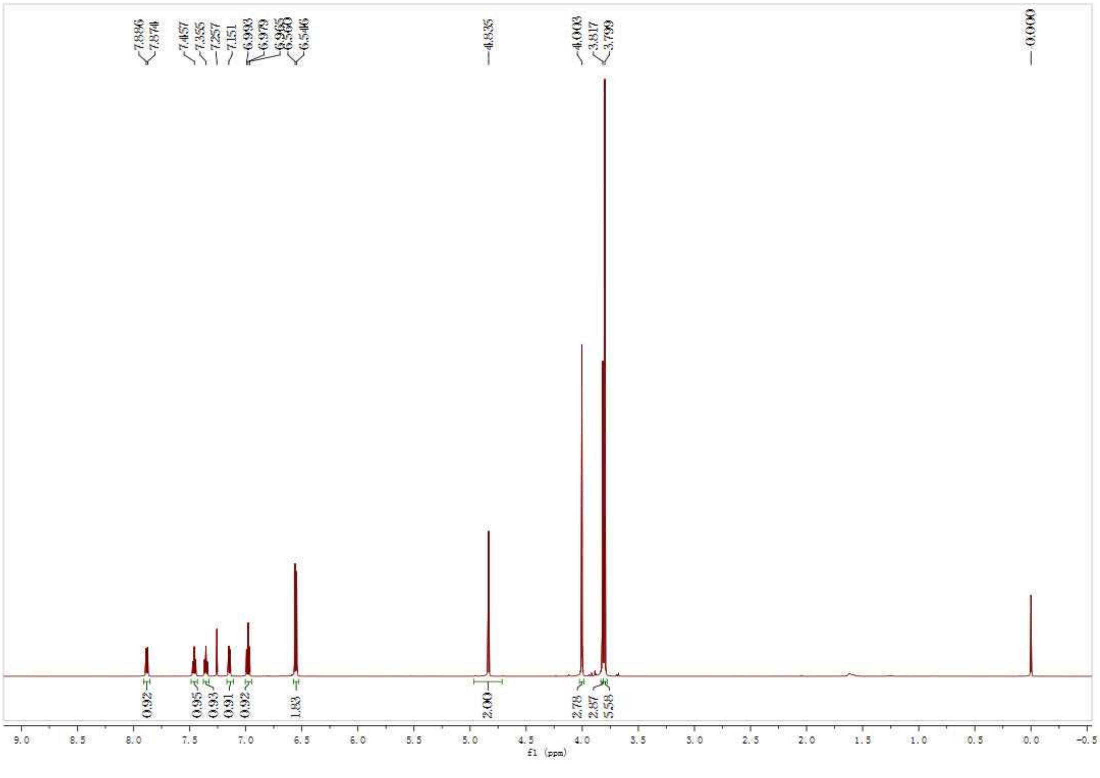
7.896
7.874
7.457
7.355
7.257
7.151
6.993
6.979
6.565
6.560
6.546
4.835
4.003
3.817
3.799
0.000
0.92
0.95
0.93
0.91
0.92
1.83
2.00
2.78
2.87
5.58
f1 (ppm)

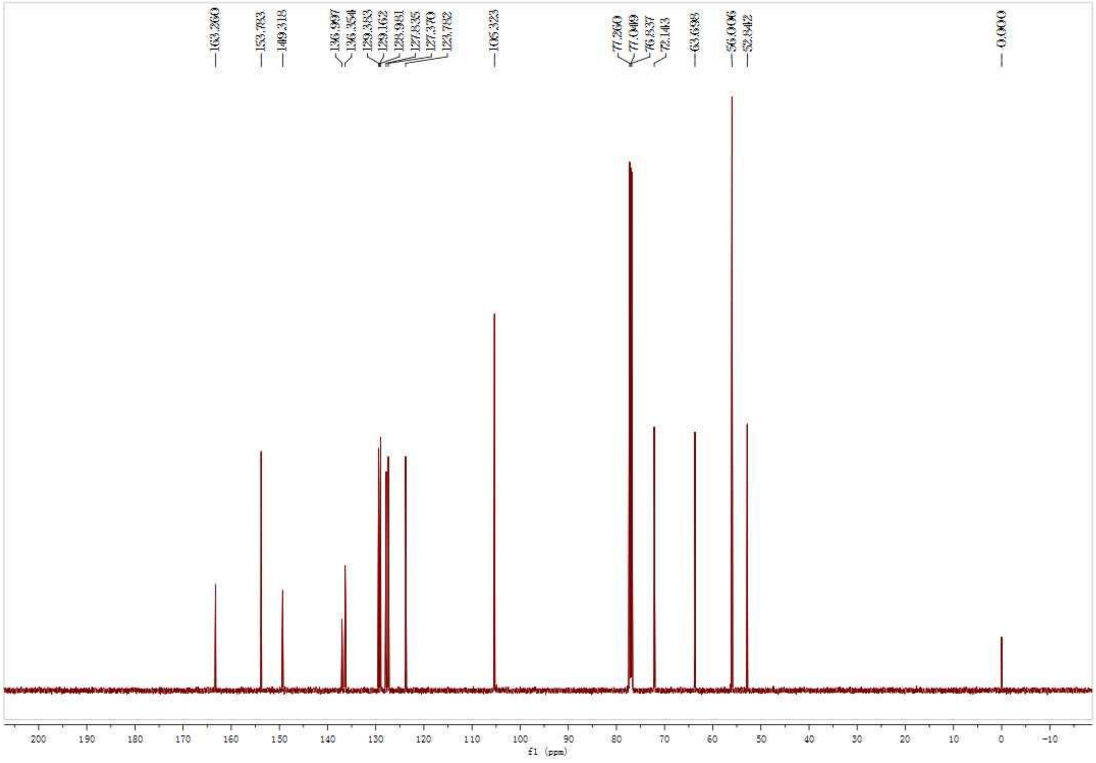

163.260
153.783
149.318
136.997
136.354
129.383
129.162
128.981
127.835
127.370
123.782
105.323
77.260
77.049
76.837
72.143
63.698
56.006
52.842
0.000
200 190 180 170 160 150 140 130 120 110 100 90 80 70 60 50 40 30 20 10 0 -10
f1 (ppm)
